# Multiview child motor development dataset for AI-driven assessment of child development

**DOI:** 10.1093/gigascience/giad039

**Published:** 2023-05-27

**Authors:** Hye Hyeon Kim, Jin Yong Kim, Bong Kyung Jang, Joo Hyun Lee, Jong Hyun Kim, Dong Hoon Lee, Hee Min Yang, Young Jo Choi, Myung Jun Sung, Tae Jun Kang, Eunah Kim, Yang Seong Oh, Jaehyun Lim, Soon-Beom Hong, Kiok Ahn, Chan Lim Park, Soon Myeong Kwon, Yu Rang Park

**Affiliations:** Department of Biomedical Systems Informatics, Yonsei University College of Medicine, Seoul 03722, Republic of Korea; Department of Biomedical Systems Informatics, Yonsei University College of Medicine, Seoul 03722, Republic of Korea; Department of Biomedical Systems Informatics, Yonsei University College of Medicine, Seoul 03722, Republic of Korea; Department of Biomedical Systems Informatics, Yonsei University College of Medicine, Seoul 03722, Republic of Korea; Department of Biomedical Systems Informatics, Yonsei University College of Medicine, Seoul 03722, Republic of Korea; Department of Biomedical Systems Informatics, Yonsei University College of Medicine, Seoul 03722, Republic of Korea; Department of Biomedical Systems Informatics, Yonsei University College of Medicine, Seoul 03722, Republic of Korea; Department of Biomedical Systems Informatics, Yonsei University College of Medicine, Seoul 03722, Republic of Korea; Department of Biomedical Systems Informatics, Yonsei University College of Medicine, Seoul 03722, Republic of Korea; MISO Info Tech Co. Ltd., Seoul 06222, Republic of Korea; Maumdri Co. Ltd., Muan-gun, Jeollanam-do 58563, Republic of Korea; Maumdri Co. Ltd., Muan-gun, Jeollanam-do 58563, Republic of Korea; Lumanlab, Inc., Seoul 05836, Republic of Korea; Division of Child and Adolescent Psychiatry, Department of Psychiatry, Seoul National University College of Medicine, Seoul 03080, Republic of Korea; Institute of Human Behavioral Medicine, Seoul National University Medical Research Center, Seoul 03080, Republic of Korea; GazziLabs, Inc., Anyang-si, Gyeonggi-do 14085, Republic of Korea; Smart Safety Laboratory Co. Ltd., Seongnam-si, Gyeonggi-do 13494, Republic of Korea; Smart Safety Laboratory Co. Ltd., Seongnam-si, Gyeonggi-do 13494, Republic of Korea; Department of Biomedical Systems Informatics, Yonsei University College of Medicine, Seoul 03722, Republic of Korea

**Keywords:** skeleton-based action recognition, children motor development, AI model

## Abstract

**Background:**

Children's motor development is a crucial tool for assessing developmental levels, identifying developmental disorders early, and taking appropriate action. Although the Korean Developmental Screening Test for Infants and Children (K-DST) can accurately assess childhood development, its dependence on parental surveys rather than reliable, professional observation limits it. This study constructed a dataset based on a skeleton of recordings of K-DST behaviors in children aged between 20 and 71 months, with and without developmental disorders. The dataset was validated using a child behavior artificial intelligence (AI) learning model to highlight its possibilities.

**Results:**

The 339 participating children were divided into 3 groups by age. We collected videos of 4 behaviors by age group from 3 different angles and extracted skeletons from them. The raw data were used to annotate labels for each image, denoting whether each child performed the behavior properly. Behaviors were selected from the K-DST's gross motor section. The number of images collected differed by age group. The original dataset underwent additional processing to improve its quality. Finally, we confirmed that our dataset can be used in the AI model with 93.94%, 87.50%, and 96.31% test accuracy for the 3 age groups in an action recognition model. Additionally, the models trained with data including multiple views showed the best performance.

**Conclusion:**

Ours is the first publicly available dataset that constitutes skeleton-based action recognition in young children according to the standardized criteria (K-DST). This dataset will enable the development of various models for developmental tests and screenings.

## Background

Motor development is essential for children's physical strength, movement, and identification of developmental difficulties. Motor development and control begin developing after birth and progress as children grow. Typically, children develop certain motor skills at a specific age; however, every child does not reach milestones at the same time [1]. Children with neurological problems, developmental delays, or disabilities may have difficulty with certain motor skills. Evaluating motor development can be a tool to assess a child's degree of development. Since a common clinical symptom of developmental milestones is not acquiring the developmental technology suitable for one's age, using simple evaluations to screen infants and toddlers with developmental problems early on [2] would be useful for planning appropriate treatment, rehabilitation, and education and improving prognoses. Additionally, early detection of developmental problems is crucial because delays can negatively affect a child's readiness to start school. Furthermore, it can cause issues with self-confidence because it is associated with the child's later achievements, such as literacy [3–5].

As a health examination project for infants and toddlers was implemented in South Korea in November 2007, the Korean Developmental Screening Test for Infants and Children (K-DST) [6] was developed to comprehensively determine the possibility of developmental disorders as well as normal development. It evaluates children's behavior, including a wide age range for preschool infants under the age of 6 (4 months to 71 months), and deals with more comprehensive developmental areas. Although the K-DST was developed specifically for Korean children, it is used globally because it is based on international standards such as the National Health Screening Program for Infants and Children [7]. Among several developmental assessment tools such as the Ages and Stages Questionnaire [8, 9], Bayley Mental Development Index [10], Bayley Scales of Infant Development, Wechsler Preschool and Primary Scale of Intelligence, and Peabody Developmental Motor Scales [11], the K-DST was selected because it can be assessed without money and has age-specific behaviors to assess motor development. In addition, recent K-DST—based research has demonstrated through national cohorts that the K-DST is a robust assessment of child development [7].

Meanwhile, the majority of existing action recognition databases have been designed for adults. There have been many studies related to children's action cognition—such as an infant action database including 18 actions extracted from Instagram and YouTube [12], action recognition including 7 actions in red, green, and blue (RGB) for children aged 6 to 11 years[13], and skeleton-driven action recognition including 6 actions for 32 children aged 6 to 9 years [14]—but there is no dataset that can be used publicly since they are all individual studies with minimal datasets or involve privacy issues.

Concerning the use of artificial intelligence (AI), various studies have evaluated children's motor functions—evaluation of cognition with physical movements [15, 16], detection of machine learning–based fine motor skills [17], and evaluation of deep learning–based children's gross motor skills [18]—but they were all AI-based, model-oriented studies. Contrastingly, this study focused on presenting a dataset of children's gross motor skills for each age group.

This study developed a new dataset for motor development in young children, from toddlers to children, using the K-DST. Although multiview recordings in previous studies [19–21] have attempted to enhance the explanatory power with more data from the combinations of anatomical feature locations from various angles, this method was selected for the following 3 additional reasons: (i) to consider the characteristics of children who are free to move and are not easy to control, (ii) to confirm the assumption that there may be a specific angle that captures a specific behavior well, and (iii) to confirm the assumption that the combination of data from certain angles can improve data learning performance results. This dataset can be used as an essential resource for the development of AI algorithms to determine children's behavior and evaluate their development.

## Methods

### Participants

All experiments were performed in accordance with the ethical principles of the Declaration of Helsinki. This study was approved by the Institutional Review Board (IRB) of Severance Hospital, Yonsei University College of Medicine, and the requirement for informed consent was waived (IRB number: 4–2021-0845). All caregivers provided written informed consent for data collection and subsequent analyses. All efforts were made to minimize the children's discomfort. The participants were children aged between 20 and 71 months from all over the country and were recruited from daycare centers, kindergartens, primary hospitals (pediatrics and adolescent medicine), and Internet communities. They were divided into 3 age groups: 20 to 35 months (group A), 36 to 53 months (group B), and 54 to 71 months (group C). The total participants included 399 children, with a sex ratio of 53/47 (male/female). Table 1 provides detailed information and sex ratios of the participants.

**Table 1:** Distribution of participants by age groups

	Total (*n* = 399)	Group A (20–35 months, *n* = 136)	Group B (36–53 months, *n* = 106)	Group C (54–71 months, *n* = 157)
Sex, *n* (%)				
Male	213 (53)	68 (50)	57 (54)	88 (56)
Female	186 (47)	68 (50)	49 (46)	69 (44)

### Type of behavior

Our dataset was collected based on the K-DST—a tool created for the accurate examination of developmental delays [22] and health management of infants and children by reflecting the characteristics of Korean infants and children. It is intended for infants and children between 4 and 71 months and includes 48 items for each age group.

Among these 48 items, core tasks were selected for each age group through consultations with 3 pediatricians and 15 child development experts based on the literature review, such as previous motor development guidelines [23, 24]. The principal criteria for selecting core tasks were (i) developmental milestones, (ii) physical and cognitive abilities, and (iii) behaviors that measure various motor skills of each age group. First, developmental milestones were identified based on a 2010 study published in *Pediatrics in Review* [24]. Second, age-appropriate physical and cognitive abilities were considered. Simple tasks were selected for younger children with limited coordination, while coordination-based tasks were adopted for older children. Third, various gross motor functions were evaluated by examining the total muscle function through various movements involving the whole body, upper body, or lower body. The representative motor development behaviors for each age group were selected to evaluate children's gross motor skills at that age. Twelve motor development tasks were defined, with 4 tasks representing each age group (Table 2).

**Table 2:** Four core motor development tasks for the 3 age groups based on the K-DST

Group	ID	Action description
Group A	1–1	Place his/her feet together and climb up the stairs one by one without holding onto the railing.
	1–2	Place his/her feet together and go down the stairs one by one without holding onto anything.
	1–3	Raise his/her arms and throw the ball over his/her head while standing.
	1–4	Stand on one foot for a second without holding onto anything.
Group B	2–1	Stand on one foot for more than three seconds without holding onto anything.
	2–2	Hop 2–3 steps on one foot.
	2–3	Put his/her feet together and make a big jump.
	2–4	Receive a big ball using both his/her arms and chest.
Group C	3–1	Stop a rolling ball with his/her feet.
	3–2	Bounce a ball on the floor once.
	3–3	Jump over a rope tied high below his/her knees.
	3–4	Jump rope once.

Based on the literature review, 18 pediatricians and experts discussed representative behaviors for each age group and selected specific actions as measurements of behavioral development.

### Experimental setup and data acquisition

**Figure 1:**
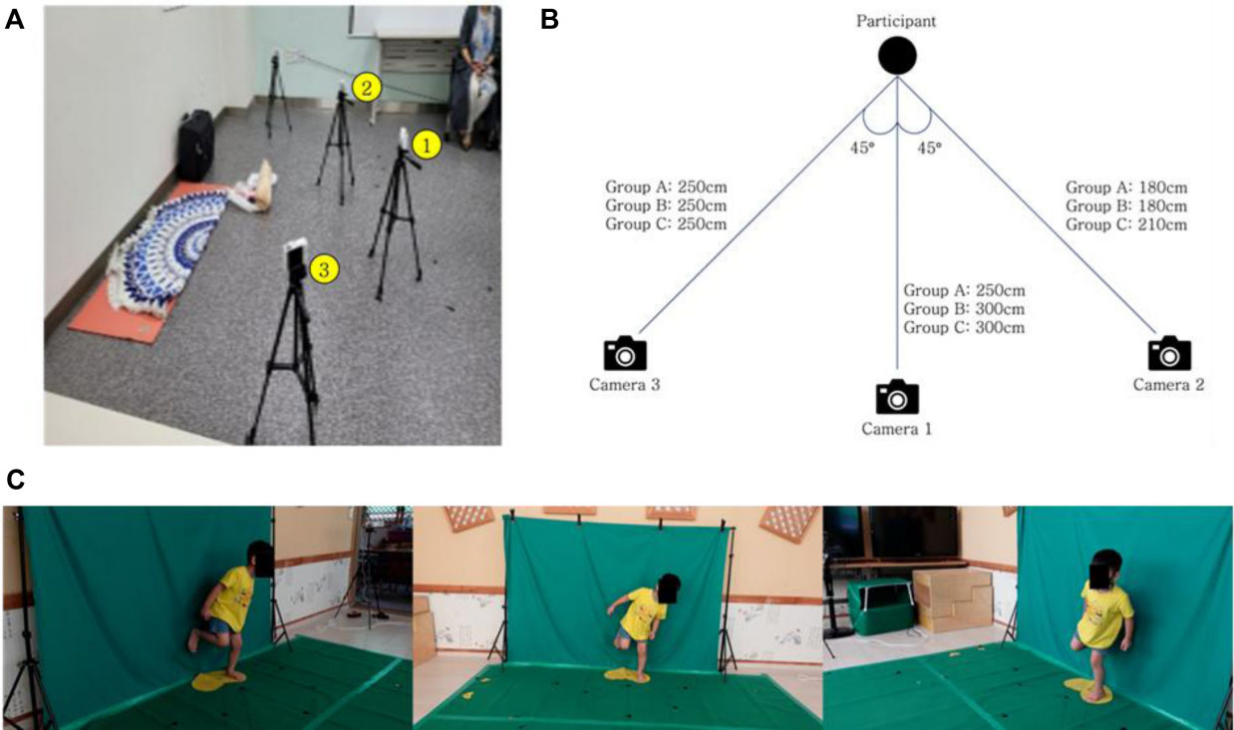
Experimental setup and data acquisition for video-based child behavior data. (A) Setting up an environment for documenting child behavior. (B) Camera angle and distance between child and camera according to age group. (C) Snapshot examples of a child's behavior video for group B behavior 1 (stand on 1 foot for more than 3 seconds without holding onto anything): view 1 (front), view 2 (right), and view 3 (left).

Participants were asked to perform 4 behaviors at least 5 to 10 times, and the behaviors were video recorded using RGB cameras. The number of trials for each behavior depended on the child's condition and cooperation. Each behavior was recorded simultaneously using 3 cameras (Fig. 1A). The distance and angle of the cameras depended on the child's age group, the details of which are described in Fig. 1B. All videos were recorded using a SONY DSC-RX100 with a resolution of 1,920 × 1,080 at 30 fps. Fig. 1C shows a portion of the videos recorded from 3 angles for behavior 1 of child B010. It represents a snapshot of a group B child's video for behavior 1 (standing on 1 foot for more than 3 seconds without holding onto anything): view 1 (front), view 2 (right), and view 3 (left). To measure the behavior of all children, the distance from the camera for each age group was defined differently based on the child with the maximum height in each age group.

### Annotation of behavior

At the labeling stage, the criteria for evaluating child development were determined based on the opinions of 12 pediatricians and child development experts (Table 3). Two evaluation processes were conducted based on these developmental evaluation criteria. In the first stage, 15 child development experts with board-certified behavior analyst certificates or equivalent experience conducted an evaluation. At this stage, behaviors were divided into 0 (bad), 1 (good), and 2 (perfect), according to each child's performance of the behavior. This evaluation method utilized a 3-point scale, which is a modification of the 4-point scale used in the K-DST. The former regards 0 (not able to do at all) and 1 (not able to do it) on the 4-point scale as 1 score (0), 2 (able to do it) as 1, and 3 (can do it well) as 2. Two or more experts simultaneously evaluated each child's behavior to increase the reliability of the evaluation results. In the second stage, pediatricians conducted an overall review based on the evaluation results of the first stage. If the results of the first evaluation stage for child behavior assessment did not match, a consensus was reached through discussion between experts, and the first evaluation was conducted again. The evaluation was conducted in 2 stages for 3 reasons. First, the opinions of pediatricians and child development experts were considered. Second, the assessments were double-checked to increase their accuracy. Third, the pediatricians’ role in the final stage was more confirmatory.

**Table 3:** Labeling criteria for child behavior

Group	Behavior ID	Labeling criteria
Group A	A01 Go up the stairs	0 (bad): He/she cannot climb up the stairs.
		1 (good): He/she can climb up the stairs but pauses a little.
		2 (perfect): He/she can climb up the stairs without difficulty.
	A02 Go down the stairs	0 (bad): He/she cannot go down the stairs.
		1 (good): He/she can go down the stairs but pauses a little.
		2 (perfect): He/she can go down the stairs without difficulty.
	A03 Throw the ball	0 (bad): He/she cannot throw the ball over his/her head.
		1 (good): He/she can throw the ball over his/her head but staggers.
		2 (perfect): He/she can throw the ball over his/her head while standing straight.
	A04 Stand on one foot	0 (bad): He/she cannot stand on one foot even for a moment.
		1 (good): He/she can stand on one foot for a second but staggers.
		2 (perfect): He/she can stand on one foot for a second without staggering.
Group B	B01 Stand on one foot	0 (bad): He/she cannot stand on one foot even for a moment.
		1 (good): He/she can stand on one foot for more than three seconds but staggers.
		2 (perfect): He/she can stand on one foot for more than three seconds without staggering.
	B02 Hop 2–3 steps	0 (bad): He/she cannot hop even once.
		1 (good): He/she can hop 2–3 steps but pauses a little.
		2 (perfect): He/she can hop 2–3 steps without difficulty.
	B03 Long jump	0 (bad): He/she cannot jump with his/her feet together.
		1 (good): He/she can jump with his/her feet together.
		2 (perfect): He/she can jump a long distance with his/her feet together.
	B04 Receive the ball	0 (bad): He/she cannot receive a ball.
		1 (good): He/she can receive a ball using both arms and chest but staggers after receiving it.
		2 (perfect): He/she can receive a ball using both arms and chest without staggering.
Group C	C01 Stop the rolling ball	0 (bad): He/she cannot stop a rolling ball with his/her foot.
		1 (good): He/she can stop a rolling ball with his/her foot.
		2 (perfect): He/she can stop a rolling ball with his/her sole.
	C02 Bounce the ball	0 (bad): He/she cannot bounce the ball on the floor at all.
		1 (good): He/she can bounce the ball on the floor once.
		2 (perfect): He/she can bounce the ball on the floor once stably.
	C03 Jump over the rope	0 (bad): He/she cannot jump over the rope tied below his/her knee level.
		1 (good): He/she can jump over the rope with a little hesitation.
		2 (perfect): He/she can jump over the rope without hesitation.
	C04 Jump rope	0 (bad): He/she cannot jump rope even once.
		1 (good): He/she can jump rope with a little hesitation.
		2 (perfect): He/she can jump rope without hesitation.

### Preprocessing of children’s behavior

**Figure 2:**
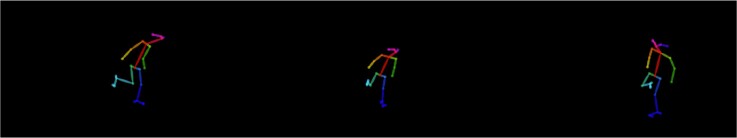
Snapshot examples of the skeleton videos extracted from the same videos in Fig. 1C. BODY_25 format was used with an output of 25 joints. These snapshots are for illustrative purposes only, and the actual data are the list of joint coordinates from entire frames. This list contains all captured joint coordinates from 1 video.

The OpenPose algorithm [25] was used to obtain human skeletal data from the RGB videos. OpenPose is a pose-estimation algorithm that extracts joint coordinates from RGB videos in 3 channels (x coordinates, y coordinates, and confidence scores). The BODY_25 format (Fig. 2) was used to obtain 25 joint coordinates per frame. Additional postprocessing was performed on the raw skeletons. First, there were missing joints in the outputs from the OpenPose algorithm; therefore, the neck joint was set as the core joint, and skeletons missing this core joint were removed because they were unreliable.

Second, although OpenPose detects multiple people in a single frame, the *n*th person at frame *t* and the *n*th person at time *t −* 1 may not be the same because they are simply listed without object identification. To solve this problem, skeletons were aligned based on the core joint (neck) [14]. Assuming that there is a neck coordinate for person 1 in frame *t*, the Euclidean distance from the neck coordinates of all people in the previous frame is calculated and connected to the closest person. Finally, the original coordinates were converted to represent the relative position based on the core joint and scaled to obtain values between 0.5 and 0.5, which can be calculated as


\begin{eqnarray*}
x &=& \left( {x/frame\_width} \right) - {x}_{neck}\\ y &=& \left( {y/frame\_height} \right) - {y}_{neck}
\end{eqnarray*}

### Evaluation for action recognition

The dataset was evaluated by training the deep learning model MS-G3D, a graph convolutional network (GCN)–based action recognition model [26]. Since only well-performed actions should be used as input data for action recognition, only data that received a score of 1 or 2 were used. The models were trained by age group, and combinations of camera views (3 angles: front, left, and right) were explored by training with data from specific views. Therefore, there were 21 models: 3 age groups and 7 view combination settings for each age group. Each model was trained with data including specific views depending on its view combination setting. Interconnections of multiviews were not considered in the models. Models were trained with the data from each view independently.

The mean lengths of data in age groups A, B, and C were 136, 167, and 87 frames, respectively. Videos were normally shorter than 300 frames based on the review of the video length histogram (see Supplementary Fig. S3). Therefore, the maximum length of input was set as 300 frames because the GCN-based action recognition models only accept inputs of the same length as recurrent neural networks (RNNs). It was padded by zero if the sample length was shorter than 300 frames and sliced to 300 frames if the sample length was longer than 300 frames.

Initially, we trained for 100 epochs to optimize the number of epochs for training. Since the models converged before 50 epochs, we trained for 50 epochs in the entire experiment (see Supplementary Fig. S2). We used an SGD optimizer with a weight decay of 0.001, a base learning rate of 0.01 (a high base learning rate is common in GCN-based action recognition model training), and a MultiStepLR learning rate scheduler with milestones (20, 30, 40), gamma 0.1. This hyperparameter setting was fixed across all models to only evaluate the effect of the combination. Additionally, the whole random seed was fixed to 100.

The dataset was split into 3 subsets based on the participants while considering the overfitting problem (see Supplement Fig. S1): training (80%), validation (10%), and testing (10%). In age group A, the training, validation, and testing sets included 4,368 samples of 104 participants, 579 samples of 14 participants, and 593 samples of 13 participants, respectively. In age group B, the training, validation, and testing sets included 2,685 samples of 80 participants, 309 samples of 11 participants, and 360 samples of 8 participants, respectively. In age group C, the training, validation, and testing sets included 5,049 samples of 125 participants, 687 samples of 17 participants, and 597 samples of 14 participants, respectively. There were 7 combinations of camera views, and each setting had the same behavior data from the same children in the training, validation, and testing sets.

## Results

### Data distribution

The data distribution of the dataset is presented in Table 4. Except for a few actions, the overall distribution was unbalanced. The distribution was most unbalanced in group C, the oldest group with the largest ratio of perfect actions. The sex ratios for each behavior were balanced in all age groups.

**Table 4:** Data distribution of the child behavior dataset

			Number (%) of videos for each label			
Group	Behavior ID	Number (%) of participants (female)	Bad	Good	Perfect	Total
Group A (*n* = 136)	A01 Climb up the stairs	136 (50)	372 (20)	303 (17)	1,130 (63)	1,805
	A02 Go down the stairs	136 (50)	400 (22)	300 (17)	1,091 (61)	1,791
	A03 Throw the ball	135 (50)	249 (14)	445 (25)	1,119 (61)	1,813
	A04 Stand on one foot	136 (50)	620 (35)	627 (35)	543 (30)	1,790
Group B (*n* = 106)	B01 Stand on one foot	98 (47)	182 (15)	504 (42)	519 (43)	1,205
	B02 Hop 2–3 steps	96 (47)	471 (45)	270 (25)	315 (30)	1,056
	B03 Long jump	103 (46)	180 (16)	213 (19)	705 (64)	1,098
	B04 Receive the ball	103 (48)	468 (36)	348 (27)	486 (37)	1,302
Group C (*n* = 157)	C01 Stop the rolling ball	154 (45)	278 (14)	415 (21)	1,315 (65)	2,008
	C02 Bounce the ball	152 (45)	204 (10)	294 (15)	1,471 (75)	1,969
	C03 Jump over the rope	148 (47)	108 (5)	264 (13)	1,648 (82)	2,020
	C04 Jump rope	137 (47)	767 (45)	534 (31)	408 (24)	1,709

### Action recognition

We explored combinations of camera views by training with data from specific views to determine the most informative one. In group A, the combination of all views showed the best performance (93.94%). In group B, the front-right combination showed the best performance (88.33%). In group C, the combination of all views showed the best performance (96.31%) (Table 5). This result indicates that if the number of views is higher, the performance is better because most of the upper ranks are combinations of multiple views. However, in the results using only a single view, the front view showed consistently good performance. This result suggests that the front view was the most informative. Therefore, in case of scarce resources, training the deep learning model using data from the front view is sufficient.

**Table 5:** Classification accuracy comparison by camera view combination

		Top-1 (%)		
No.	Camera view combination	Group A	Group B	Group C
1	Front, left, right	93.94	87.50	96.31
2	Front, right	93.69	88.33	94.22
3	Front, left	92.17	85.83	95.23
4	Right, left	91.67	85.83	96.23
5	Front	92.93	82.50	90.95
6	Right	92.93	77.50	92.96
7	Left	85.35	85.83	91.46

Additionally, the confusion matrices of 3-view models and single-view models were obtained (see Supplementary Fig. S4). The confusion matrices show that the diagonal of the 3-view model's matrix had higher values than the front-view model's matrix. In other words, 3-view models showed better performance than single-view models.

## Conclusion

The dataset presented in this study consisted of young children divided into 3 age groups, based on the K-DST, performing 4 representative behaviors for each age group that were recorded and collected from 3 different angles. To represent child development datasets, we first defined core behaviors in the child development process by age group through active discussions with a group of child development experts, including pediatricians. Second, we established evaluation criteria in 3 stages for each behavior for clear and reliable evaluation. The data were collected from 399 children. Each video of child behavior was manually labeled using a 3-point scale for the evaluation of motor development created by 15 developmental assessment experts and 3 pediatricians. As a result of applying a deep learning–based action recognition model to verify the quality of the developed dataset, data collected from 2 or more directions performed better than individual directions. Our dataset is the first publicly accessible dataset that enables the identification and evaluation of young children's actions and motor development based on their skeletons. This study emphasized gross motor skills based on a previous study [7] that found gross motor skills to have more accuracy than fine motor skills in the K-DST for children's motor skill evaluation. However, other previous studies [3, 27] have shown that fine motor skills are also valuable in evaluating children's motor skills. In our future work, we will compare fine and gross motor skill evaluations to enhance the accuracy of child development evaluation. Additionally, the dataset will be extended to include children with and without developmental disabilities. It can be utilized to develop early diagnostic prediction models using AI techniques such as machine learning. Since the dataset provided in this study includes scores, it can be used to develop a model for predicting scores. Furthermore, it can be utilized as the basis for developing screening tools for children's quantitative motor development levels (body maturity). Moreover, it was found that utilizing the multiview data had positive effects on the model training. In our future work, we will measure the effect of multiple views by combining multiple data as an extended concept of multiple data utilization.

## Data Availability

All collected data are available in our GitHub repository [28]. All supporting data and materials are also available in the *GigaScience* GigaDB database [29].

## Additional Files

**Supplementary Fig. S1**. Loss graphs of each model. The yellow lines display the valid loss, while the blue lines show the training loss. Our experiment consisted of 21 models, encompassing 3 age groups and 7 view settings per age. Each column presents graphs for a particular age group, ordered as follows: age group A, age group B, and age group C. Each row displays graphs for a specific view setting, arranged as follows: View123, View12, View13, View23, View1, View2, and View3. Across all settings, the training loss and valid loss converged simultaneously, and none of the 21 models exhibited any signs of loss explosion, indicating the absence of overfitting during the model training process.

**Supplementary Fig. S2**. Training graphs of each model. The yellow lines indicate train loss, and the blue lines represent train accuracy. Our experiment consisted of a total of 21 models, comprising 3 age groups and 7 view settings per age group. Each column in the graphs corresponds to 1 age group, ordered as follows: age group A, age group B, and age group C. Furthermore, each row displays the graphs for 1 view combination, following the sequence of View123, View12, View13, View23, View1, View2, and View3. The graphs exhibit train accuracy and train loss for 100 epochs, revealing that the models converged before 50 epochs of training, despite the absence of pretraining.

**Supplementary Fig. S3**. Video frame length histograms for each age group. The x-axis represents the frame length of each video, while the y-axis indicates the number of videos. The histograms indicate that most videos were less than 300 frames in length; consequently, we designated the maximum frame length for model inputs as 300 frames.

**Supplementary Fig. S4**. Confusion matrices of each age group model predictions for the testing set. (A), (B), and (C) in the left column are for single (front) view models, while (D), (E), and (F) in the right column are for 3-view models. The confusion matrices indicate that models trained with 3-view data outperformed those trained with front-view data. Specifically, the diagonal values of the 3-view model's matrix were higher compared to those of the front-view model, indicating superior performance of the 3-view models over the single-view models.

giad039_GIGA-D-22-00210_Original_Submission

giad039_GIGA-D-22-00210_Revision_1

giad039_Response_to_Reviewer_Comments_Original_Submission

giad039_Reviewer_1_Report_Original_Submission Ashwin Ramesh Babu, Ph.D. -- 11/15/2022 Reviewed

giad039_Reviewer_1_Report_Revision_1 Ashwin Ramesh Babu, Ph.D. -- 3/21/2023 Reviewed

giad039_Reviewer_2_Report_Original_Submission Lei Ma -- 1/16/2023 Reviewed

giad039_Reviewer_3_Report_Original_Submission Tracy Anne Hammond -- 1/24/2023 Reviewed

giad039_Supplemental_File

## Availability of Source Code and Requirements

Project name: Multiview child motor development dataset for AI-driven assessment of child development

- Project homepage: [30]
- Operating system(s): Linux
- Programming language: Python3
- Other requirements: PyTorch> = 1.2.0, pyyaml, tensorboardX, tqdm, glob
- License: MIT license
- RRID: SCR_023552

## Abbreviations

AI: artificial intelligence; GCN: graph convolutional network; IRB: institutional review board; K-DST: Korean Developmental Screening Test for Infants and Children; RGB: red, green, and blue.

## Authors’ contributions

Conceptualization: H.H.K., J.Y.K., and Y.R.P. Methodology: H.H.K., J.Y.K., and Y.R.P. Data collection: E.K. Y.S.O., J.H.K., and S.B.H. Data cropping and parsing: B.K.J., J.H.L., J.H.K., D.H.L., H.M.Y., Y.J.C., M.J.S. Data curation: K.A.A. Data evaluation: C.L.P. and S.M.K. Annotation validation: E.K. Y.S.O., J.H.K., and S.B.H. Writing: H.H.K., J.Y.K., and Y.R.P. AI modeling and validation: J.Y.K. Supervision: Y.R.P. Project administration: T.J.K. All authors have read and agreed to the published version of the manuscript.

## Competing interests

The authors declare that they have no competing interests.

## Funding

This research was supported by a grant for the R&D project, funded by the National Center for Mental Health (grant number: MHER22A01).

This article used datasets from machine learning data collection projects funded by the Ministry of Science & ICT and the National Information Society Agency (NIA, S. Korea, grant number: 13-08-0-130-133-2000–2033).
