# Supplementary material for: Multiview child motor development dataset for AI-driven assessment of child development
Source: Gigascience. 2023 May 27;12:giad039. doi: 10.1093/gigascience/giad039 (PMC10220505; doi:10.1093/gigascience/giad039)
Supplement: giad039_GIGA-D-22-00210_Original_Submission [file giad039_giga-d-22-00210_original_submission.pdf]

# GigaScience


# Multi-view child motor development dataset for AI-driven assessment of child development

--Manuscript Draft--

| | |
|---|---|
| **Manuscript Number:** | GIGA-D-22-00210 |
| **Full Title:** | Multi-view child motor development dataset for AI-driven assessment of child development |
| **Article Type:** | Data Note |
| **Funding Information:** | National Center for Mental Health (MHER22A01) — Ms. Yu Rang Park |
| **Abstract:** | Background<br><br>Children’s motor development is a crucial tool for assessing developmental levels, identifying developmental disorders early, and taking appropriate action. Although the Korean Developmental Screening Test for Infants and Children (K-DST) can accurately assess childhood development, its dependence on parental surveys rather than reliable, professional observation limits it. This study constructs a dataset based on a skeleton of recordings of K-DST behaviors in children with and without developmental disorders between 20 and 71 months. We validated the dataset using a child behavior artificial intelligence (AI) learning model to highlight its possibilities.<br><br>Results<br><br>The 339 participating children were divided into three groups by age. We collected videos of four behaviors by age group at three different angles and extracted skeletons from them. The raw data were used to annotate labels for each image, denoting whether each child performed the behavior properly. Behaviors were selected from the K-DST’s gross motor section. The number of images collected differed by age group. The original dataset underwent additional processing to improve its quality. Finally, we confirmed that our dataset can be used in the AI model with 90%, 87.67%, and 95.45% test accuracy by age group in an action recognition model. Additionally, the combination of values measured for each angle performed the best.<br><br>Conclusion<br><br>Ours is the first publicly available dataset that constitutes skeleton-based action recognition in young children according to standardized criteria (K-DST). This dataset will enable the development of various models for developmental tests and screenings. |
| **Corresponding Author:** | Yu Rang Park, Ph.D.<br><br>KOREA, REPUBLIC OF |
| **Corresponding Author Secondary Information:** | |
| **Corresponding Author's Institution:** | |
| **Corresponding Author's Secondary Institution:** | |
| **First Author:** | Hye Hyeon Kim, PhD |
| **First Author Secondary Information:** | |
| **Order of Authors:** | Hye Hyeon Kim, PhD |
| | Jin Yong Kim, BS |
| | Bong Kyung Jang, BS |
| | Joo Hyun Lee, BS |
| | |


Powered by Editorial Manager® and ProduXion Manager® from Aries Systems Corporation

| | |
|---|---|
| | Jong Hyun Kim, BS |
| | Dong Hoon Lee, BS |
| | Hee Min Yang, BS |
| | Young Jo Choi, BS |
| | Myung Jun Sung, BS |
| | Tae Jun Kang, BS |
| | Eunah Kim, MA |
| | Yang Seong Oh, MS |
| | Jaehyun Lim, MD, PhD |
| | Soon-Beom Hong, MD, PhD |
| | Kiok Ahn, PhD |
| | Chan Lim Park, MS |
| | Soon Myeong Kwon, BS |
| | Yu Rang Park, PhD |
| **Order of Authors Secondary Information:** | |


| | |
|---|---|
| **Additional Information:** | |
| **Question** | **Response** |
| Are you submitting this manuscript to a special series or article collection? | No |
| **Experimental design and statistics**<br><br>Full details of the experimental design and statistical methods used should be given in the Methods section, as detailed in our Minimum Standards Reporting Checklist. Information essential to interpreting the data presented should be made available in the figure legends.<br><br>Have you included all the information requested in your manuscript? | Yes |
| **Resources**<br><br>A description of all resources used, including antibodies, cell lines, animals and software tools, with enough information to allow them to be uniquely identified, should be included in the Methods section. Authors are strongly encouraged to cite Research Resource Identifiers (RRIDs) for antibodies, model organisms and tools, where possible. | Yes |

| | |
|---|---|
| Have you included the information requested as detailed in our Minimum Standards Reporting Checklist? | |
| **Availability of data and materials**<br><br>All datasets and code on which the conclusions of the paper rely must be either included in your submission or deposited in publicly available repositories (where available and ethically appropriate), referencing such data using a unique identifier in the references and in the "Availability of Data and Materials" section of your manuscript.<br><br>Have you have met the above requirement as detailed in our Minimum Standards Reporting Checklist? | Yes |

Powered by Editorial Manager® and ProduXion Manager® from Aries Systems Corporation

Click here to access/download;Manuscript;manuscript_nia_data_v2.docx



# Multi-view child motor development dataset for AI-driven assessment of child development

Hye Hyeon Kim[1#], PhD; Jin Yong Kim[1#], BS; Bong Kyung Jang[1], BS; Joo Hyun Lee[1], BS; Jong Hyun Kim[1], BS; Dong Hoon Lee[1], BS; Hee Min Yang[1], BS; Young Jo Choi[1], BS; Myung Jun Sung[1], BS; Tae Jun Kang[2], BS; Eunah Kim[3], MA; Yang Seong Oh[3], MS; Jaehyun Lim[4], MD, PhD; Soon-Beom Hong[5, 6], MD, PhD; Kiok Ahn[7], PhD; Chan Lim Park[8], MS; Soon Myeong Kwon[8], BS; Yu Rang Park[1*], PhD

[1] Department of Biomedical Systems Informatics, Yonsei University College of Medicine, Seoul, Republic of Korea

[2] MISO Info Tech Co. Ltd., Seoul, Republic of Korea

[3] Maumdri Co. Ltd., Seoul, Republic of Korea

[4] Lumanlab, Inc., Seoul, Republic of Korea

[5] Division of Child and Adolescent Psychiatry, Department of Psychiatry, Seoul National University College of Medicine, Seoul, Republic of Korea

[6] Institute of Human Behavioral Medicine, Seoul National University Medical Research Center, Seoul, Republic of Korea

[7] GazziLabs, Inc., Seoul, Republic of Korea

[8] Smart Safety Laboratory Co. Ltd., Seoul, Republic of Korea

[#] HH Kim and JY Kim contributed equally to this paper

***Corresponding author:** Yu Rang Park, PhD

Department of Biomedical System Informatics, Yonsei University College of Medicine

50-1 Yonsei-ro, Seodaemun-gu, Seoul 03722, Korea

Phone: +82-2228-2493

Tel: 82-2-2228-2493, E-mail: yurangpark@yuhs.ac

**Abstract**

**Background:** Children's motor development is a crucial tool for assessing developmental levels, identifying developmental disorders early, and taking appropriate action. Although the Korean Developmental Screening Test for Infants and Children (K-DST) can accurately assess childhood development, its dependence on parental surveys rather than reliable, professional observation limits it. This study constructs a dataset based on a skeleton of recordings of K-DST behaviors in children with and without developmental disorders between 20 and 71 months. We validated the dataset using a child behavior artificial intelligence (AI) learning model to highlight its possibilities.

**Results:** The 339 participating children were divided into three groups by age. We collected videos of four behaviors by age group at three different angles and extracted skeletons from them. The raw data were used to annotate labels for each image, denoting whether each child performed the behavior properly. Behaviors were selected from the K-DST's gross motor section. The number of images collected differed by age group. The original dataset underwent additional processing to improve its quality. Finally, we confirmed that our dataset can be used in the AI model with 90%, 87.67%, and 95.45% test accuracy by age group in an action recognition model. Additionally, the combination of values measured for each angle performed the best.

**Conclusion:** Ours is the first publicly available dataset that constitutes skeleton-based action recognition in young children according to standardized criteria (K-DST). This dataset will enable the development of various models for developmental tests and screenings.


**Keywords:** skeleton-based action recognition; children motor development; AI model

**1. Background**

Motor development is important as it is essential for children’s physical strength, movement, and identifying developmental problems. Motor development and control begin developing after birth and progress as children grow. Typically, children develop certain motor skills at a specific age, but not every child reaches milestones at the same time [1]. Children with neurological problems, developmental delays, or disabilities may have difficulty with certain motor skills. Evaluating motor development can be a tool used to assess a child’s degree of development. Since the most common clinical symptom of developmental disability is not acquiring developmental technology suitable for one's age, using simple evaluations to screen infants and toddlers with developmental problems early on [2] would be useful for planning appropriate treatment, rehabilitation, and education and for improving prognoses.

As a health examination project for infants and toddlers was implemented in South Korea in November 2007, the Korean Developmental Screening Test for Infants and Children (K-DST) [3] was developed to comprehensively determine the possibility of developmental disorders as well as normal development. It evaluates children's behavior, including a wide age range for preschool infants under the age of six (4 months to 71 months), and deals with more comprehensive developmental areas. Although the KDST was developed specifically for Korean children, it is also globally applicable because it is based on international standards such as the National Health Screening Program for Infants and Children [4].

Meanwhile, the majority of existing action recognition databases are designed for adults. There have been many studies related to children’s action cognition—such as an infant action database including 18 actions extracted from Instagram and YouTube [5], action recognition

including seven actions in RGB for children ages 6–11 [6], and skeleton driven action recognition including six actions for 32 children ages 6–9 [7]—but there is no dataset that can publicly be used since they are all individual studies with minimal datasets or involve privacy issues.

We developed a new dataset for motor development in young children, from toddlers to children, using the K-DST. Although multi-view recordings in previous studies [8-10] have intended to enhance explanatory power with more data from the combinations of anatomical feature locations from various angles, we selected this method for the following three additional reasons: 1) we needed to consider the characteristics of children who are free to move and are not easy to control, 2) we wanted to confirm the assumption that there may be a specific angle that captures a specific behavior well, and 3) we also wanted to confirm the assumption that the combination of data from certain angles can improve data learning performance results.

This dataset can be used as an essential resource for the development of artificial intelligence algorithms to determine children's behavior and evaluate their development.

## 2. Methods

### 2.1 Participants

All experiments were performed in accordance with the ethical principles of the Declaration of Helsinki. This study was approved by the Institutional Review Board of Severance Hospital, Yonsei University College of Medicine, and the requirement for informed consent was waived (IRB number: 4-2021-0845). All caregivers provided written

informed consent for the sample collection and subsequent analyses. All efforts were made to minimize the suffering and discomfort of the children. The participants were children between 20 and 71 months of age from all over the country and were recruited from daycare centers, kindergartens, primary hospitals (pediatrics and adolescent medicine), and Internet communities. They were divided into three age groups: 20-35 months (Group A), 36-53 months (Group B), and 54-71 months (Group C). A total of 399 children participated, with a sex ratio of 53:47. Detailed numbers and sex ratios of the participants are shown in Table 1.

**Table 1** Distribution of participants by age groups.

| | Total (n=399) | Group A (20-35 months, n=136) | Group B (36-53 months, n=106) | Group C (54-71 months, n=157) |
|---|---|---|---|---|
| Sex (n, %) | | | | |
| Male | 213 (53%) | 68 (50%) | 57 (54%) | 88 (56%) |
| Female | 186 (47%) | 68 (50%) | 49 (46%) | 69 (44%) |

**2.2 Type of behavior**

Our dataset was collected based on the K-DST, which is a tool created for the accurate examination of developmental delay [11] and health management of infants and children by reflecting the characteristics of Korean infants and children. It is intended for infants and children between 4 and 71 months and provides 48 items for each age group.

Among these 48 items, we selected core tasks for each age group through consultation with three pediatricians and 15 child behavior development experts with literature reviews, such as previous motor development guidelines [12, 13]. The representative motor development behaviors for each age group were selected to evaluate the children’s gross motor skills at

that age. Twelve motor development tasks were defined, with four tasks representing each age group (Table 2).

Based on literature reviews, 15 pediatricians and experts discussed representative behaviors for each age group and selected specific actions as measurements of behavioral development.

**Table 2** Four core motor development tasks for four age groups based on the K-DST.

| Group | ID | Action Description |
|---|---|---|
| Group A | 1-1 | Place his/her feet together and climb up the stairs one by one without holding on to the railing. |
| | 1-2 | Place his/her feet together and go down the stairs one by one without holding on to anything. |
| | 1-3 | Raise his/her arms and throw the ball over his/her head while standing. |
| | 1-4 | Stand on one foot for a second without holding onto anything. |
| Group B | 2-1 | Stand on one foot for more than three seconds without holding onto anything. |
| | 2-2 | Hop 2–3 steps on one foot. |
| | 2-3 | Put his/her feet together and make a big jump. |
| | 2-4 | Receive a big ball using both his/her arms and chest. |
| Group C | 3-1 | Stop a rolling ball with his/her feet. |
| | 3-2 | Bounce a ball on the floor once. |
| | 3-3 | Jump over a rope tied high below his/her knees. |
| | 3-4 | Jump rope once. |

## 2.3 Experimental setup and data acquisition

We had participants perform four behaviors at least five to ten times, and the behaviors were video-recorded using RGB cameras. The number of trials for each behavior depended on the child's condition and cooperation. Each behavior was recorded simultaneously from three cameras (Figure 1A). The distance and angle of the cameras depended on the child's age group, and the details are described in Figure 1B. All videos were collected using a SONY DSC-RX100 with a resolution of 1920x1080, fps 30. Figure 1C shows a portion of the videos collected from three angles for Behavior 1 of Child B010. These are snapshot examples of a

child's behavior video for Group B's Behavior 1 (standing on one foot for more than three seconds without holding onto anything): view 1 (middle), view 2 (right), and view 3 (left).

## 2.4 Annotation of behavior

At the labeling stage, the criteria for evaluating child development were determined by receiving opinions from 12 pediatricians and child development experts (Table 3). Two evaluation processes were conducted based on these developmental evaluation criteria. The first step was an evaluation conducted by 15 child development evaluation experts with board-certified behavior analyst certificates or equivalent experience. At this stage, behaviors were divided into 0 (Bad), 1 (Good), and 2 (Perfect), according to each child's performance of the behavior. Two or more experts simultaneously evaluated each child's behavior to increase the reliability of the evaluation results. In the second stage, pediatricians conducted an overall review based on the evaluation results of the first stage. If the results of the first stage of evaluation did not match, a consensus was arrived at through close consultation between experts, and the first evaluation was conducted again.

**Table 3** Labeling criteria for child behavior.

| Group | Behavior ID | Labeling criteria |
|---|---|---|
| Group A | A01<br>Go up the stairs | 0 (bad): He/She cannot climb up the stairs.<br>1 (good): He/She can climb up the stairs but pauses a little.<br>2 (perfect): He/She can climb up the stairs without difficulty. |
| | A02<br>Go down the stairs | 0 (bad): He/She cannot go down the stairs.<br>1 (good): He/She can go down the stairs but pauses a little.<br>2 (perfect): He/She can go down the stairs without difficulty. |
| | A03<br>Throw the ball | 0 (bad): He/She cannot throw the ball over his/her head.<br>1 (good): He/She can throw the ball over his/her head but staggers.<br>2 (perfect): He/She can throw the ball over his/her head while standing straight. |
| | A04 | 0 (bad): He/She cannot stand on one foot even for a moment. |

| | | |
|---|---|---|
| | Stand on one foot | 1 (good): He/She can stand on one foot for a second but staggers.<br>2 (perfect): He/She can stand on one foot for a second without staggering. |
| Group B | B01<br>Stand on one foot | 0 (bad): He/She cannot stand on one foot even for a moment.<br>1 (good): He/She can stand on one foot for more than three seconds but staggers.<br>2 (perfect): He/She can stand on one foot for more than three seconds without staggering. |
| | B02<br>Hop 2-3 steps | 0 (bad): He/She cannot hop even once.<br>1 (good): He/She can hop 2–3 steps but pauses a little.<br>2 (perfect): He/She can hop 2–3 steps without difficulty. |
| | B03<br>Long jump | 0 (bad): He/She cannot jump with his/her feet together.<br>1 (good): He/She can jump with his/her feet together.<br>2 (perfect): He/She can jump a long distance with his/her feet together. |
| | B04<br>Receive the ball | 0 (bad): He/She cannot receive a ball.<br>1 (good): He/She can receive a ball using both arms and chest but staggers after receiving.<br>2 (perfect): He/She can receive a ball using both arms and chest without staggering. |
| Group C | C01<br>Stop the rolling ball | 0 (bad): He/She cannot stop a rolling ball with his/her foot.<br>1 (good): He/She can stop a rolling ball with his/her foot.<br>2 (perfect): He/She can stop a rolling ball with his/her sole. |
| | C02<br>Bounce the ball | 0 (bad): He/She cannot bounce the ball on the floor at all.<br>1 (good): He/She can bounce the ball on the floor once.<br>2 (perfect): He/She can bounce the ball on the floor once stably. |
| | C03<br>Jump over the rope | 0 (bad): He/She cannot jump over the rope tied below his/her knees.<br>1 (good): He/She can jump over the rope with a little hesitation.<br>2 (perfect): He/She can jump over the rope without hesitation. |
| | C04<br>Jump rope | 0 (bad): He/She cannot jump rope even once.<br>1 (good): He/She can jump rope with a little hesitation.<br>2 (perfect): He/She can jump rope without hesitation. |

### 2.5 Preprocessing of children's behavior

We used the OpenPose algorithm [14] to obtain human skeletal data from the RGB videos. OpenPose is a pose-estimation algorithm that extracts joint coordinates from RGB videos in three channels (x coordinates, y coordinates, and confidence scores). We used the BODY_25 format (Figure 2) to obtain 25 joint coordinates per frame. Additional post-processing was

performed on the raw skeletons. First, there were missing joints in the outputs from the OpenPose algorithm, so we set the neck joint as the core joint and removed skeletons missing this core joint because they were unreliable.

Second, although OpenPose detects multiple people in a single frame, the *n-th* person at frame *t* and the *n-th* person at time *t-1* may not be the same person because they are simply listed without object identification. To solve this problem, skeletons were aligned based on the core joint (neck) [7]. Assuming that there is a neck coordinate for person 1 in frame *t*, the Euclidean distance from the neck coordinates of all people in the previous frame is calculated and connected to the closest person. Finally, we converted the original coordinates to represent the relative position based on the core joint and scaled them to have values between –0.5 and 0.5, which can be calculated as

$$x = (x/frame\_width) - x_{neck}$$

$$y = (y/frame\_height) - y_{neck}$$

### 2.6 Evaluation for action recognition

We evaluated our dataset by training the deep learning model MS-G3D, the GCN-based action recognition model [15]. For action recognition, since only well-performed actions should be used as input data, only data that received a score of one or two were used. The models were trained by age group, and we explored combinations of camera views (three angles: front, x, and y) by training with data from specific views.

We trained all models for 50 epochs, using an SGD optimizer with weight decay 0.001, base learning rate 0.01(a high base learning rate is common in GCN-based action recognition model training), and a MultiStepLR learning rate scheduler with milestones, gamma 0.1. This

hyperparameter setting was fixed across all models to evaluate only the effect of the combination. Additionally, the whole random seed was fixed to 100.

The dataset was split into three subsets: training (80%), validation (10%), and testing (10%). There were seven combinations of camera views, and each setting had the same behavior data from the same children, except for camera views in the training and testing sets.

## 3. Results

### 3.1 Data distribution

The data distribution of the dataset is presented in Table 4. Except for a few actions, the overall distribution was unbalanced. The distribution was most unbalanced in Group C, the oldest group, with the largest ratio of perfect actions. The sex ratios for each behavior were balanced in all age groups.

**Table 4** Data distribution of child behavior dataset.

| Group | Behavior ID | Number of participants (female %) | Number of videos for each label | | | |
|---|---|---|---|---|---|---|
| | | | Bad (n, %) | Good (n, %) | Perfect (n, %) | Total |
| Group A (n =136) | A01 Climb up the stairs | 136 (50%) | 372 (20%) | 303 (17%) | 1130 (63%) | 1805 |
| | A02 Go down the stairs | 136 (50%) | 400 (22%) | 300 (17%) | 1091 (61%) | 1791 |
| | A03 Throw the ball | 135 (50%) | 249 (14%) | 445 (25%) | 1119 (61%) | 1813 |
| | A04 Stand on one foot | 136 (50%) | 620 (35%) | 627 (35%) | 543 (30%) | 1790 |
| Group B (n =106) | B01 Stand on one foot | 98 (47%) | 182 (15%) | 504 (42%) | 519 (43%) | 1205 |
| | B02 Hop 2-3 steps | 96 (47%) | 471 (45%) | 270 (25%) | 315 (30%) | 1056 |
| | B03 Long jump | 103 (46%) | 180 (16%) | 213 (19%) | 705 (64%) | 1098 |
| | B04 | 103 (48%) | 468 | 348 | 486 | 1302 |

| | | | | | | |
|---|---|---|---|---|---|---|
| | Receive the ball | | (36%) | (27%) | (37%) | |
| Group C (n =157) | C01 Stop the rolling ball | 154 (45%) | 278 (14%) | 415 (21%) | 1315 (65%) | 2008 |
| | C02 Bounce the ball | 152 (45%) | 204 (10%) | 294 (15%) | 1471 (75%) | 1969 |
| | C03 Jump over the rope | 148 (47%) | 108 (5%) | 264 (13%) | 1648 (82%) | 2020 |
| | C04 Jump rope | 137 (47%) | 767 (45%) | 534 (31%) | 408 (24%) | 1709 |

### 3.2 Action recognition

We explored combinations of camera views by training with data from specific views to determine which was the most informative. In Group A, the combination of all views showed the best performance (93.94%); in Group B, the front-right combination showed the best performance (88.33%); and in Group C, the combination of all views showed the best performance (96.31%) (Table 5). This result means that if the number of views is higher, the performance is better because most of the upper ranks are combinations of multiple views. However, in the results using only a single view, the front view showed consistently good performance. This result suggests that the front view was the most informative. Therefore, if resources are lacking, training the deep learning model using data from the front view is sufficient.

**Table 5** Classification accuracy comparison by camera view combination.

| **No.** | **Camera view combination** | **Top-1 (%)** | | |
|---|---|---|---|---|
| | | **Group A** | **Group B** | **Group C** |
| **1** | Front, Left, Right | 93.94 | 87.50 | 96.31 |
| **2** | Front, Right | 93.69 | 88.33 | 94.22 |
| **3** | Front, Left | 92.17 | 85.83 | 95.23 |
| **4** | Right, Left | 91.67 | 85.83 | 96.23 |
| **5** | Front | 92.93 | 82.50 | 90.95 |
| **6** | Right | 92.93 | 77.50 | 92.96 |
| **7** | Left | 85.35 | 85.83 | 91.46 |

## Conclusion

The dataset presented in this study consisted of young children divided into three age groups based on the K-DST performing four representative behaviors for each age group that were recorded and collected from three different angles. To represent child development datasets, we first defined core behaviors in the child development process by age group through active discussions with a group of child development experts, including pediatricians. Second, we established evaluation criteria in three stages for each behavior for clear and reliable evaluation. The data were collected from 399 children. Each video of child behavior was manually labeled using a 3-point scale for the evaluation of motor development developed by 15 developmental assessment experts and three pediatricians. As a result of applying a deep learning-based behavior detection model to verify the quality of the developed dataset, data collected from two or more directions performed better than individual directions. Intending to contribute to the field, our dataset is the first publicly accessible dataset that enables the identification and evaluation of young children's actions and motor development based on their skeletons. In future work, this dataset can be used as a behavioral biomarker that can distinguish between children with normal and delayed development and will also be used as an early diagnosis prediction model using AI techniques such as machine learning. This dataset can also be used to develop screening tools for children’s quantitative motor development levels (body maturity).

## List of abbreviations

K-DST, Korean Developmental Screening Test for Infants and Children; GCN, Graph Convolutional Networks; RGB, red-green-blue

## Competing interests

The authors declare that they have no competing interests.

## Funding

This research was supported by a grant of the R&D project, funded by the National Center for Mental Health (grant number: MHER22A01).

## Availability of Data and Materials

All collected dataset is available from Github: https://github.com/DigitalHealthcareLab/22ActionRecognitionTool

## Authors' contributions

Conceptualization: HH Kim, JY Kim, and YR Park; Methodology: HH Kim, JY Kim, and YR Park; Data Collection: E Kim, YS Oh, JH Kim, and SB Hong; Data Cropping and Parsing: BK Jang, JH Lee, JH Kim, DH Lee, HM Yang, YJ Choi, MJ Sung; Data Curation: KA Ahn; Data Evaluation: CL Park and SM Kwon; Annotation Validation: E Kim, YS Oh, JH Kim, and SB Hong; Writing: HH Kim, JY Kim, and YR Park; AI Modeling and Validation: JY Kim; Supervision: YR Park; Project Administration: TJ Kang; All authors have read and agreed to the published version of the manuscript.

## References

1. Group, W.H.O.M.G.R.S., *WHO Motor Development Study: windows of achievement for six gross motor development milestones.* Acta Paediatr Suppl, 2006. **450**: p. 86-95.
2. Karmel, B.Z., et al., *Early medical and behavioral characteristics of NICU infants later classified with ASD.* Pediatrics, 2010. **126**(3): p. 457-67.
3. Jang, C.H., et al., *Clinical Usefulness of the Korean Developmental Screening Test (K-DST) for Developmental Delays.* Ann Rehabil Med, 2019. **43**(4): p. 490-496.
4. Chung, H.J., et al., *Development of the Korean Developmental Screening Test for Infants and Children (K-DST).* Clin Exp Pediatr, 2020. **63**(11): p. 438-446.
5. Balasathiya, S.S., S.M.M. Roomi, and B. Sathyabama. *Infant Action Database: A Benchmark for Infant Action Recognition in Uncontrolled condition*. in *Journal of Physics: Conference Series*. 2021. IOP Publishing.
6. Turarova, A., et al., *Child Action Recognition in RGB and RGB-D Data.* Hri'20: Companion of the 2020 Acm/Ieee International Conference on Human-Robot Interaction, 2020: p. 491-492.
7. Silva, V., et al., *Skeleton Driven Action Recognition Using an Image-Based Spatial-Temporal Representation and Convolution Neural Network.* Sensors (Basel), 2021. **21**(13).
8. Nicora, E., et al., *The MoCA dataset, kinematic and multi-view visual streams of fine-grained cooking actions.* Sci Data, 2020. **7**(1): p. 432.
9. Tang, J., et al., *Improved multi-view privileged support vector machine.* Neural Netw, 2018. **106**: p. 96-109.
10. Wang, Y., et al., *Discriminative Multi-View Dynamic Image Fusion for Cross-View 3-D Action Recognition.* IEEE Trans Neural Netw Learn Syst, 2021. **PP**.
11. Shin, S.M., *Should we regularly evaluate the neurodevelopmental status of moderate and late preterm infants?* Clin Exp Pediatr, 2020. **63**(6): p. 217-218.
12. McWilliams, C., et al., *Best-practice guidelines for physical activity at child care.* Pediatrics, 2009. **124**(6): p. 1650-9.
13. Gerber, R.J., T. Wilks, and C. Erdie-Lalena, *Developmental milestones: motor development.* Pediatr Rev, 2010. **31**(7): p. 267-76; quiz 277.
14. Cao, Z., et al., *OpenPose: Realtime Multi-Person 2D Pose Estimation Using Part Affinity Fields.* IEEE Trans Pattern Anal Mach Intell, 2021. **43**(1): p. 172-186.
15. Liu, Z., et al. *Disentangling and unifying graph convolutions for skeleton-based action recognition*. in *Proceedings of the IEEE/CVF conference on computer vision and pattern recognition*. 2020.

**Figure Titles**

**Figure 1** Experimental setup and data acquisition for video-based child behavior data. (A) Setting up an environment for documenting child behavior. (B) Camera angle and distance between child and camera according to age group. (C) Snapshot examples of a child's behavior video for Group B Behavior 1 (stand on one foot for more than three seconds without holding onto anything): view 1 (middle), view 2 (right), and view 3 (left).

**Figure 2.** Snapshot examples of the skeleton videos extracted from the same videos in Figure 1C. BODY_25 format was used, and it has an output of 25 joints. These snapshots are for illustrative purposes only, and the actual data is compiled as a list of joint coordinates.

Figure Click here to access/download;Figure;Figure_1.docx

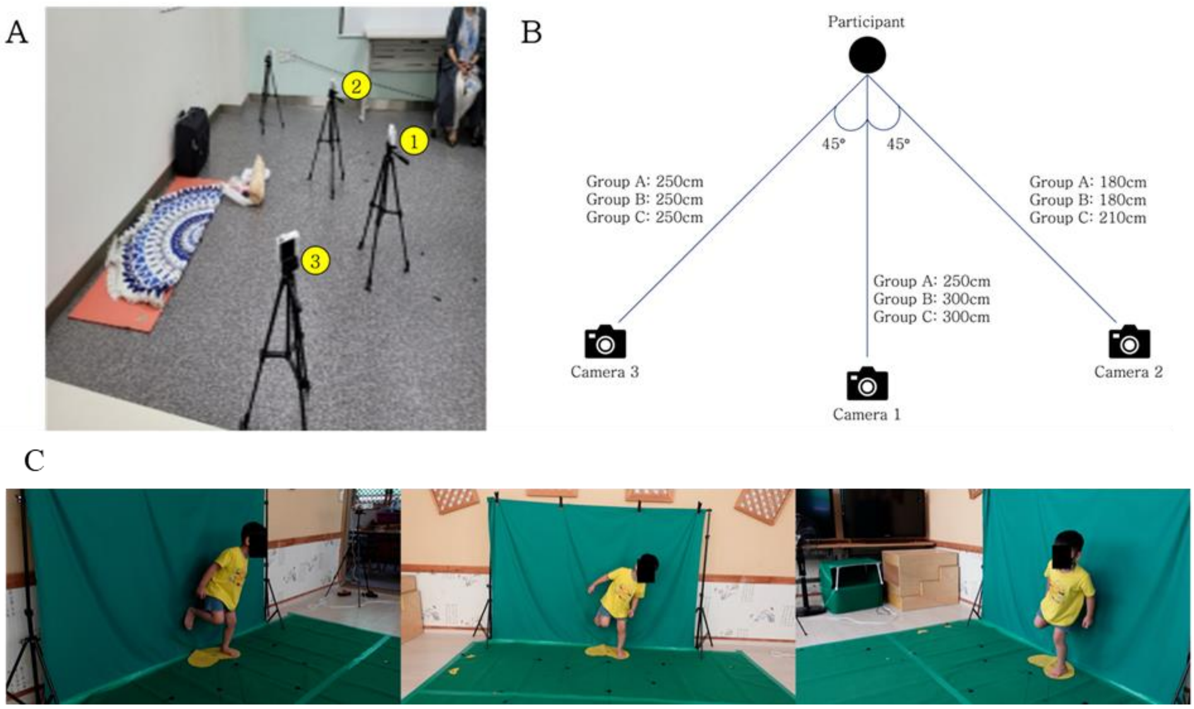

Figure Click here to access/download;Figure;Figure_2.docx

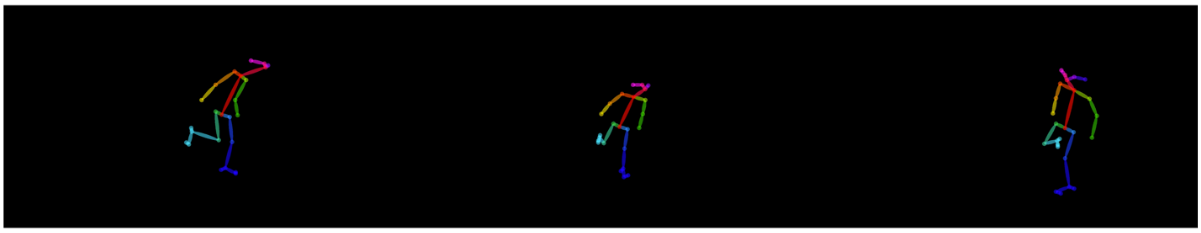

Click here to access/download
**Supplementary Material**
GigaDBUploadForm_Final.xlsx
