# Supplementary material for: Multiview child motor development dataset for AI-driven assessment of child development
Source: Gigascience. 2023 May 27;12:giad039. doi: 10.1093/gigascience/giad039 (PMC10220505; doi:10.1093/gigascience/giad039)
Supplement: giad039_GIGA-D-22-00210_Revision_1 [file giad039_giga-d-22-00210_revision_1.pdf]

GigaScience

Multi-view child motor development dataset for AI-driven assessment of child development

--Manuscript Draft--

**Manuscript Number:**	GIGA-D-22-00210R1
**Full Title:**	Multi-view child motor development dataset for AI-driven assessment of child development
**Article Type:**	Data Note
**Funding Information:**	National Center for Mental Health (MHER22A01) — Ms. Yu Rang Park
**Abstract:**	Background: Children’s motor development is a crucial tool for assessing developmental levels, identifying developmental disorders early, and taking appropriate action. Although the Korean Developmental Screening Test for Infants and Children (K-DST) can accurately assess childhood development, its dependence on parental surveys rather than reliable, professional observation limits it. This study constructed a dataset based on a skeleton of recordings of K-DST behaviors in children aged between 20 and 71 months, with and without developmental disorders. The dataset was validated using a child behavior artificial intelligence (AI) learning model to highlight its possibilities.<br>Results: The 339 participating children were divided into three groups by age. We collected videos of four behaviors by age group from three different angles and extracted skeletons from them. The raw data were used to annotate labels for each image, denoting whether each child performed the behavior properly. Behaviors were selected from the K-DST’s gross motor section. The number of images collected differed by age group. The original dataset underwent additional processing to improve its quality. Finally, we confirmed that our dataset can be used in the AI model with 93.94%, 87.50%, and 96.31% test accuracy for the three age groups in an action recognition model. Additionally, the models trained with data including multiple views showed the best performance.<br>Conclusion: Ours is the first publicly available dataset that constitutes skeleton-based action recognition in young children according to the standardized criteria (K-DST). This dataset will enable the development of various models for developmental tests and screenings.
**Corresponding Author:**	Yu Rang Park, Ph.D.<br><br>KOREA, REPUBLIC OF
**Corresponding Author Secondary Information:**	
**Corresponding Author's Institution:**	
**Corresponding Author's Secondary Institution:**	
**First Author:**	Hye Hyeon Kim, PhD
**First Author Secondary Information:**	
**Order of Authors:**	Hye Hyeon Kim, PhD
	Jin Yong Kim, BS
	Bong Kyung Jang, BS
	Joo Hyun Lee, BS
	Jong Hyun Kim, BS
	Dong Hoon Lee, BS
	Hee Min Yang, BS

Powered by Editorial Manager® and ProduXion Manager® from Aries Systems Corporation

	Young Jo Choi, BS
	Myung Jun Sung, BS
	Tae Jun Kang, BS
	Eunah Kim, MA
	Yang Seong Oh, MS
	Jaehyun Lim, MD, PhD
	Soon-Beom Hong, MD, PhD
	Kiok Ahn, PhD
	Chan Lim Park, MS
	Soon Myeong Kwon, BS
	Yu Rang Park, PhD
**Order of Authors Secondary Information:**	

Response to Reviewers:

Rebuttal Letter

March 15, 2023
Travis Sanchez,
Editors-in-Chief
JMIR Public Health and Surveillance

Dear Editor-in-Chief:

My colleagues and I would like to re-submit our revised manuscript entitled, “Multi-view child motor development dataset for AI-driven assessment of child development,” for reconsideration as an original article in the GigaScience. This is the revised version of the previous manuscript (GIGA-D-22-00210).

First, we would like to thank the reviewers of the GigaScience for taking the time and effort to review our manuscript. The reviewers’ comments were constructive and raised important and valuable points that provided us with some new insights about the study and required more clarification in the revised manuscript. Over the past few weeks, we have tried our best to improve our paper by attempting to fulfill all the reviewers’ requirements. We have prepared detailed responses to the reviewers’ comments. The modifications in the revised manuscript and supplemental material according to the reviewers’ comments have been highlighted in blue characters. Additionally, we have thoroughly checked the grammatical errors in our manuscript again.

All authors have critically read and agreed to the re-submission of this manuscript. The paper is not under consideration elsewhere, and none of the paper’s content has been published previously. In addition, no author of this manuscript has any relationship with the industry associated with the content of the manuscript.
Thank you in advance for the review.
Best regards,

Yu Rang Park, PhD
Associate Professor
Department of Biomedical Systems Informatics, Yonsei University College of Medicine, Yonsei University College of Medicine, 50-1 Yonsei-ro, Seodaemungu, Seoul, South Korea
Phone: +82-10-5240-3434
E-mail: yurangpark@yuhs.ac

Reviewer #1:

Concerns:
The authors claim that this is the first dataset of this kind to evaluate cognitive and motor functionality using AI.The paper is weak in its related works.
There are multiple papers that propose various tasks to assess embodied cognition in children
using machine and deep learning.

Eg.
1. An automated assessment system for embodied cognition in children: from motion data to executive functioning
2. A multi-modal system to assess cognition in children from their physical movements
-focused to assess cognition with one physical movement
3. Motor assessment using the NIH Toolbox
4. Assessment of motor functioning in the preschool period
5. Detecting Children's Fine Motor Skill Development using Machine Learning
-focused on children's fine motor to implement classifiers (Fine model)
6. Deep learning assessment of child gross-motor

Specifically, papers 1,2,5,6 uses deep learning to evaluate motor functions in children.
Response: Thank you for informing us about the existing assessment tools that were lacking in our preliminary survey. We wanted to emphasize that our study is the first study to provide public access to the dataset. None of the previous studies mentioned in your comment have released their dataset. However, since motor skill evaluation using AI is valuable, the mentioned studies associated with the evaluation of motor functions were compared with our study in the Background section as follows.

"Concerning the use of artificial intelligence, various studies have evaluated children's motor functions—evaluation of cognition with physical movements[15,16], detection of machine learning-based fine motor skills[17], and evaluation of deep learning-based children's gross motor skills[18]—but they were all AI-based, model-oriented studies. Contrastingly, this study focused on presenting a dataset of children's gross motor skills for each age group."

Data collection:
The dataset consists of kids with healthy and specific conditions.Was there any correlation done between the kids with conditions and their performance to evaluate theeffectiveness of the task?
Response: Our dataset only included motor functioning in healthy children. Therefore, we did not need to perform correlations. However, it is meaningful to include children with specific conditions. We will consider it in our future work. We have added the following description in the Conclusion section.

"Additionally, the dataset will be extended to include children with and without developmental disabilities. It can be utilized to develop early diagnostic prediction models using AI techniques such as machine learning."

Question related to training.
How were the data split for the training and validation? Were they split based on the participants or based on samples?In computer vision, splitting the data based on samples might cause overfitting.
This is because,if a same kid is present in both training and validation set, its considered to memorize.

Response: Thank you for pointing out the unclear explanation. We split the data in 8(train):1(valid):1(test) ratio for the training and validation based on participants considering the overfitting problem as you have mentioned. We also obtained the loss curves after training, which showed that there was no overfitting problem. We have added loss graphs to Figure S1 of the supplement to show that there were no overfitting problems. Additionally, we have included a detailed description of the data split in Section 2.6 Evaluation for action recognition of the Method as follows.

"The dataset was split into three subsets based on the participants while considering

the overfitting problem (see Supplement Figure S1): training (80%), validation (10%), and testing (10%). In age group A, the training, validation, and testing sets included 4,368 samples of 104 participants, 579 samples of 14 participants, and 593 samples of 13 participants, respectively. In age group B, the training, validation, and testing sets included 2,685 videos of 80 participants, 309 samples of 11 participants, and 360 samples of 8 participants, respectively. In age group C, the training, validation, and testing sets included 5049 samples of 125 participants, 687 samples of 17 participants, and 597 samples of 14 participants, respectively."

Training for 50 epochs seems too less for a video action recognition model. Were there any pre-training done?
Response: Thank you for your valuable question about our experiment setting. Although it was not mentioned in the manuscript, we trained for 100 epochs before training for 50 epochs because we found that our model had sufficiently converged before that point. Therefore, we did not pre-train the models. We have added the training graph with 100 epochs, including train accuracy and train loss to Figure S2 of the supplement for a better explanation of the epoch setting. We have described it in Section 2.6 Evaluation for action recognition of the Method as follows.

"Initially, we trained for 100 epochs to optimize the number of epochs for training. Since the models converged before 50 epochs, we trained for 50 epochs in the entire experiment (see Supplement Figure S2)."

So the Action recognition model can predict what action is being performed or even the score for the action?
Response: Thank you for seeking clarification on the action recognition model. The action recognition model can only predict actions being performed but not the score of the actions. Since the dataset disclosed by us includes scores (it is labeled), it can be used to develop a model for predicting scores in future work. We have added the following explanation to the Conclusion section.

"Since the dataset provided in this study includes scores, it can be used to develop a model for predicting scores. Furthermore, it can be utilized as the basis for developing screening tools for children's quantitative motor development levels (body maturity)."

How long was each recording? was a single recording split into multiple actions for the training and validation purpose?More details regarding the Model used, is required.
Response: Thank you for pointing out the insufficient description. The mean video length of age group A was 136 frames, the mean length of age group B was 167 frames, and the mean video length of age group C was 87 frames. We also obtained histograms of video lengths; video lengths were normally under 300 frames. Therefore, we set the maximum length of input data by 300 frames because the GCN-based action models that we used only accept inputs of the same length as RNNs. We padded it by zero if the sample length was shorter than 300 frames and sliced it to 300 frames if the sample length was longer than 300 frames. We did not split a single recording into multiple actions because each of our recordings contained only one trial for one kind of action. For the readers of this paper, we have added video length histograms to the supplement. Additionally, we have added the explanation of this process to Section 2.6 Evaluation for action recognition of the Method as follows.

"The mean length of data in age groups A, B, and C were 136, 167, and 87 frames, respectively. Videos were normally shorter than 300 frames based on the review of the video length histogram (see Supplement Figure S3). Therefore, the maximum length of input was set as 300 frames because the GCN-based action recognition models only accept inputs of the same length as RNNs. It was padded by zero if the sample length was shorter than 300 frames and sliced to 300 frames if the sample length was longer than 300 frames."

As this is a classification problem, a confusion matrix is required to get complete insight on the model performance.

Powered by Editorial Manager® and ProduXion Manager® from Aries Systems Corporation

Response: Thank you for letting us know that our results were insufficient. We have added the confusion matrices of three view models to Figure S4 of the supplement to provide a complete insight into the model performance, as per your valuable comment. The confusion matrix showed that models trained with three-view data were better than the models trained with front-view data only. We have added the explanation of confusion matrices to Section 3.2 Action recognition of the Result section as follows.

"Additionally, the confusion matrices of three-view models and single-view models were obtained (see Supplement Figure S4). The confusion matrices show that the diagonal of the three-view model's matrix had higher values than the front-view model's matrix. In other words, three-view models showed better performance than single-view models."

Reviewer #2:

The study constructed a new dataset based on a skeleton of recordings of K-DST behaviors in children and validated the dataset using artificial intelligence model. In general, I think this work presents good research and has strong practical value. But this paper still has number of limitations. I suggest the authors make the following revisions.

Abstract
-p.2, Results: Why are the test accuracy results (…90%, 87.67%, and 95.45%...) inconsistent with the Section 3.2 Action recognition (Table 5)?
Response: Thank you for pointing our mistake. We have made corrections for consistent results in the Abstract.

Methods
-p.4: The acronym of "IRB" should be preceded by the full name.
Response: Thank you. We have added the full name of IRB as follows.

"This study was approved by the Institutional Review Board of Severance Hospital, Yonsei University College of Medicine, and the requirement for informed consent was waived (Institutional Review Board [IRB] number: 4-2021-0845)."

-p.5, section 2.2 Type of behavior, the second paragraph: What is the basis or principle for selecting the representative motor development behaviors for each age group? Please explain this.

Response: Thank you for your valuable comment.
As described in the methodology section of this paper, four core tasks were selected for each age group from a total of 48 GMS tasks provided by the K-DST. These core tasks were chosen by three pediatricians and 15 child behavior development experts based on the following three specific criteria. First, developmental milestones of each age group were considered in the selection of motor developmental behaviors, with the typical age at which these milestones are achieved identified from a 2010 study [24]. Second, physical and cognitive abilities were considered when selecting the core behaviors. Simple tasks, such as standing on one foot, were chosen for younger age groups with limited coordination abilities, whereas older age groups were given tasks that focused on coordination, such as stopping a rolling ball with one foot. Finally, actions that could measure various gross muscle functions were selected for each age group, including actions that involved the movement of the entire body, upper body, or lower body. Overall, the selection of representative motor development behaviors for each age group in this study was based on a combination of developmental milestones, physical and cognitive abilities, and actions that could measure various gross motor development functions.
For the readers of this paper, an explanation of this process has been added to Section 2.2 Type of behavior of the Method as follows.

"The principal criteria for selecting core tasks were: 1) developmental milestones, 2) physical and cognitive abilities, and 3) behaviors that measure various motor skills of

Powered by Editorial Manager® and ProduXion Manager® from Aries Systems Corporation

each age group. First, developmental milestones were identified based on a 2010 pediatric review study [24]. Second, age-appropriate physical and cognitive abilities were considered. Simple tasks were selected for younger children with limited coordination, while coordination-based tasks were adopted for older children. Third, various gross motor functions were evaluated by examining the total muscle function through various movements involving the whole body, upper body, or lower body.”

-p.6, the title of Table 2: "…for four age groups…" should be "…for three age groups…".
Response: Thank you for pointing our mistake. We have revised it as per your correction.

-p.6, section 2.3 Experimental setup and data acquisition, the first paragraph: Why is the distance parameter different in different age groups (Figure 1B)? Is there any standard for setting this parameter?
Response: Thank you for pointing out the insufficient explanation. The distance from the camera for each age group was defined based on the child with the maximum height in the age group to measure the behavior of all children. We have added the following description in Section 2.3 Experimental setup and data acquisition of the Method section.

“To measure the behavior of all children, the distance from the camera for each age group was defined differently based on the child with the maximum height in each age group.”

-p.6, section 2.4 Annotation of behavior, the first paragraph: What is the role and content of the second stage review?
Response: Thank you for pointing out the insufficient explanation. We asked the same questions at both stages, but the pediatrician played a more confirmatory role. We also wanted to consider all perspectives of child development experts and pediatricians and increase the accuracy of evaluations with double reviews. We have added the following description in Section 2.4 Annotation of behavior of the Method section.

“The evaluation was conducted in two stages for three reasons. First, the opinions of pediatricians and child development experts were considered. Second, the assessments were double-checked to increase their accuracy. Third, the pediatricians’ role in the final stage was more confirmatory.”

Format
-Table 1-5: These tables can be presented in the format of "three-line table" because it is simple in form and easy to read.
-The format of the table should be consistent, such as the alignment, bold font (The header of Table 5).
-The text of this paper should be aligned at both ends.
Response: Thank you for detailed comments. We have made revisions based on your suggestions.

Reviewer #3: This paper presents a dataset of infants and children's activities used as criteria for assessing childhood development based on the Korean Development Screening Test (K-DST). The dataset comprises 399 infants and children. The age range is from 20 months to 71 months. The participants were grouped into three age groups: 20-35, 36-53, and 54-71 (younger, middle-aged, and older). The authors developed four criteria for assessing the motor skills of each group using the K-DST, following consultation with experts in the field.For data collection, three cameras were used to record the behavior of the participants 3-5 times. The footage was then annotated by experts to evaluate the behavior. After preprocessing the data, the dataset was analyzed using the deep learning model MS-G3D and the GCN-based action recognition model. The overall results demonstrated fairly good performance. This paper claims that the dataset is a valuable resource for creating AI algorithms that assess children's behavior and track their development.

This is an interesting paper on the collection and analysis of a dataset focused on motor assessment of young children using camera-recorded gross motor activities. It is motivated by the need for easy, unbiased diagnostics that can be used to assess if children are suffering from motor delay, and by lack of activity data for young children, as current activity recognition models mostly focus on adults. In addition to the dataset, the paper includes an analysis of using pose estimation from the video to categorize children into year-and-a-half age groups.Overall, I like how this paper is written. It is concise, mostly clear, and covers a lot of the key points. I do have a few suggestions, which are described below.

In the first paragraph, there is mention that the most common clinical symptom of developmental disability is delayed acquisition of developmental technology. I think this sentence could be improved in a few ways:

- First, the phrasing is a little confusing to me because it sounds like the issue is a lack of access to devices. I would use the term "developmental milestones" as stated earlier in the paper.
Response: Thank you for your insightful comment. We have revised it in the Background section as per your suggestion.

“Since a common clinical symptom of developmental milestones …”

- Second, I don't see strong citation support for this being the most common clinical symptom; the current citation is more an analysis of the demographics and specific factors that were observed in children who later received ASD diagnoses. I would agree that missed milestones is certainly the most prominent symptom since many parents pay attention to these, and the assessments are largely based on tasks associated with specific milestones. That said, there are other symptoms which could signal disability or delay, so I'd suggest including a reference that it is most common or adjusting the phrasing to be "a very common clinical symptom" or similar. It is a small change but could make the language more approachable to both experts and laypersons.
Response: We agree that there have been deficiencies as we have only emphasized ASD diagnoses. Based on your suggestion, we have revised the sentence.

- Third, on the point of citations, I think you could frame it as the need for catching developmental issues early because of (a) the association between motor development and later achievements like literacy and (b) the negative impact of delays on ability to start school and corresponding self-confidence issues. Below are a few citations you might want to consider to support these points; there are a number of relevant research studies which could further support the motivation if desired.

Duncan, Greg J., Chantelle J. Dowsett, Amy Claessens, Katherine Magnuson, Aletha C. Huston, Pamela Klebanov, Linda S. Pagani et al. "School readiness and later achievement." Developmental psychology 43, no. 6 (2007): 1428.

Burkam, David T., Laura LoGerfo, Doug Ready, and Valerie E. Lee. "The differential effects of repeating kindergarten." Journal of Education for Students Placed at Risk 12, no. 2 (2007): 103-136.

Broughman, Stephen P., and Mary R. Rollefson. "Children who enter kindergarten late or repeat kindergarten: Their characteristics and later school performance." Education Statistics Quarterly 2, no. 3 (2000): 21.

Suggate, Sebastian, Eva Pufke, and HeidrunStoeger. "Do fine motor skills contribute to early reading development?." Journal of Research in Reading 41, no. 1 (2018): 1-19.

Grissmer, David, Kevin J. Grimm, Sophie M. Aiyer, William M. Murrah, and Joel S. Steele. "Fine motor skills and early comprehension of the world: two new school readiness indicators." Developmental psychology 46, no. 5 (2010): 1008.

Response: Thank you for providing references to help us modify the need for early

Powered by Editorial Manager® and ProduXion Manager® from Aries Systems Corporation

detection of developmental difficulties in a more understandable way. We have added the following sentences in the Introduction based on your recommended references.

“Additionally, early detection of developmental problems is crucial because delays can negatively affect a child’s readiness to start school. Furthermore, it can cause issues with self-confidence because it is associated with the child’s later achievements, such as literacy [3-5].”

About those last two: it's worth noting that they highlight the value of fine motor skill assessments in particular versus gross motor skill ones. While fine motor assessment is important, and I'd hope to see more on that in the future, I think there is definitely room to argue for the value of any motor assessments.I'm sure there is research support for that, which would be good to consider since this paper predominantly focuses on gross motor assessment.Along those same lines, it would be worth adding a small discussion about gross vs. fine motor skills. This wouldn't have to be extensive, but it could be part of the motivation for why the gross motor portion of the KDST is used as the baseline as opposed to fine motor tasks. I'm not very familiar with the KDST, so perhaps it doesn't have as much of a fine motor section. I mention it because some of the research talks about fine motor being more critical for certain tasks, but as I said above, I do believe the gross motor assessment to be just as important with the right motivation!
Response: Thank you for highlighting the significance of assessing fine motor skills. Although we have concentrated on gross motor skills based on the result of the previous study that gross motor skills have more accuracy than fine motor in the K-DST, the importance of considering fine motor will be helpful in our future work. We have added the following in the Conclusion section.

“This study emphasized gross motor skills based on a previous study [7] that found the gross motor to have more accuracy than the fine motor in the K-DST for children’s motor skill evaluation. However, other previous studies [3, 27] have shown that fine motor skills are also valuable in evaluating children’s motor skills. In our future work, we will compare fine and gross motor skill evaluations to enhance the accuracy of child development evaluation.”

Given the locality of the study, it's reasonable to use the KDST. If you wish, you could mention that other general assessments exist, like the Ages and Sages Questionnaire or the Bailey Mental Development Index. I don't feel it's necessary, but even a small mention that there are such assessments before introducing the KDST would be fine.
Response: Thank you for informing us about the existing assessment tools that were insufficient in our preliminary survey. We have added the following sentences in the Background section.

“Among several general development assessment tools for children such as such as the Ages and Stages Questionnaire [8,9], Bayley Mental Development Index [10], Bayley Scales of Infant Development, Wechsler Preschool and Primary Scale of Intelligence, and Peabody Developmental Motor Scales [11], the K-DST was selected because it can be assessed without money and has age-specific behaviors to assess motor development. In addition, recent K-DST-based research has demonstrated through national cohorts that the K-DST is a robust assessment of child development [7].”

How were the age groups selected for the dataset? That is, what was the process behind making Group A cover ages 20 to 35 months, Group B 36 to 53 months, and so on? I like these groupings and believe they align fairly well with key developmental stages, but I'm curious about the origin of the selection: something from the KDST, attempt to make the groups similarly sized, or something else. I often see these types of datasets use year-level scales, but I like the choice you made here.
Response: Precisely, our research team was concerned about recruiting an adequate number of children to evaluate gross motor skills through the AI model if the age group was too narrow. To address this concern, the K-DST target age of 4 to 71 months was divided into four age groups (4 to 19 months, 10 to 35 months, 36 to 53 months, and

Powered by Editorial Manager® and ProduXion Manager® from Aries Systems Corporation

54 to 71 months). However, due to the COVID-19 pandemic in Korea, fewer children were recruited than expected, especially in the youngest age group (4 to 19 months), with only 27 participants. Consequently, behavioral evaluation by AI models was difficult, and concerns about privacy violations arose if a group with too few participants was disclosed. Therefore, the age group of 4 to 19 months was excluded from the study to ensure that the size of each group was comparable and to avoid potential privacy concerns.

Likewise, the scoring mechanism of 0, 1, and 2: is this based on the standard scoring approach the experts would use? This 3-point scale seems reasonable, but it would be worth including the motivation for the selection versus something like a binary or 5-point scale.
Response: Thank you for your valuable comment. In this study, we presented a 3-point scale for gross motor scoring that modified the scoring criteria of the K-DST. The original K-DST evaluation scale is a 4-point scale. According to this scale, 0, 1, 2, and 3 points indicate not able to do at all, not able to do it, able to do it, and can do it well, respectively. Considering the original K-DST scoring system, there are almost no behaviors that received a score of 0 in our dataset. In order to match the similarity of data size between scores, we combined the original K-DST scores 0 and 1 and treated them as 0 points (bad), 2 as 1 (good), and 3 as 2 (perfect).
For the readers of this paper, an explanation of this process has been added to Section 2.4 Annotation of behavior of the Method as follows.

"This evaluation method utilized a 3-point scale, which is a modification of the 4-point scale used in the K-DST. The former regards 0 (not able to do at all) and 1 (not able to do it) in the 4-point scale as one score (0), 2 (able to do it) as 1, and 3 (can do it well) as 2."

Table 5 uses Front, Left, and Right while the text uses front, x, and y. This could be remedied in the text by mentioning, even parenthetically, which is left and which is right after the angles of x and y are first mentioned.
Response: Thank you for correcting our mistake. We have revised it as "Front, Left, and Right" for consistency.

I'm not completely familiar with the method applied, so it would improve the readability to better explain how the combination of view angels worked. Based on the description, I'm imagining that one angle is fed frame-by-frame in into the machine learning system using pose-estimation coordinates. When combining views, are these concatenated so that all poses are sent at the same time, or are they interlaced in some manner?
Response: Thank you for your detailed question about our experiment. We did not consider how multi views were interlaced; however, in the results, the models trained with multi-view data showed better performance than models trained with only single-view data. It means that utilizing data from multi views had positive effects on the model training. However, it is essential to measure multi view's actual effect, which we plan to measure in our future work. We have added the explanation of this future work to the Conclusion section as follows.

"Moreover, it was found that utilizing the multi-view data had positive effects on the model training. In our future work, we will measure the effect of multiple views by combining multiple data as an extended concept of multiple data utilization."

Figure 2 caption mentions they are compiled into a list of joint coordinates, but it's not clear how this scale with multiple poses since the paper only discusses selecting a single pose from a frame.

Response: Thank you for pointing out the insufficient explanation. The list of coordinates mentioned in Figure 2 contains all the captured joint coordinates from only one view and not all the multi views. Initially, we had input the whole view at once and trained the models for each view separately as a feature extractor and combined the

Powered by Editorial Manager® and ProduXion Manager® from Aries Systems Corporation

output features extracted from each model by a fully connected layer. However, the model did not converge and had overfitting problems. Therefore, we trained the models with data from each view independently. We have added the training graphs of the combination model in the Figure S5 of the supplement. For the readers of this paper, we have added a detailed explanation to Section 2.6 Evaluation for action recognition of the Method as follows.

“Therefore, there were 21 models: three age groups and seven view combination settings for each age group. Each model was trained with data including specific views depending on its view combination setting. Interconnections of multi views were not considered in the models. Models were trained with the data from each view independently.”

The results look good! I'd say the combination of all views worked best. I'm not sure if I'd say front individually is the most informative since it seems like the combination is what helps most. In any case, it's an impressive result, and even at the year-and-a-half level of granularity, it's a good showing of a possible motor skill diagnostic, mostly on gross motor tasks.

Given how much modern computer science paper bibliographies have expanded, I'm a little surprised to see comparatively few citations. That said, I don't see an issue as long as it's enough to support everything stated or argued in the paper.

In conclusion, I really liked this paper! It's very direct and a pleasure to read. I do have a few suggestions above for ways make it more clear. I wouldn't say a lot needs to be changed since the paper is appropriately concise, just some clarifications and extra citations.

References

3.Grissmer, D., et al., Fine motor skills and early comprehension of the world: two new school readiness indicators. Dev Psychol, 2010. 46(5): p. 1008-17.
4.Kamphorst, E., et al., Emerging School Readiness Profiles: Motor Skills Matter for Cognitive- and Non-cognitive First Grade School Outcomes. Front Psychol, 2021. 12: p. 759480.
5.Duncan, G.J., et al., School readiness and later achievement. Dev Psychol, 2007. 43(6): p. 1428-1446.
7.Chung, H.J., et al., Development of the Korean Developmental Screening Test for Infants and Children (K-DST). Clin Exp Pediatr, 2020. 63(11): p. 438-446.
8.Romero Otalvaro, A.M., et al., ASQ-3: Validation of the Ages and Stages Questionnaire for the detection of neurodevelopmental disorders in Argentine children. Arch Argent Pediatr, 2018. 116(1): p. 7-13.
9.Veldman, S.L., et al., Prevalence and risk factors of gross motor delay in pre-schoolers. J Paediatr Child Health, 2020. 56(4): p. 571-576.
10.Bos, A.F., Bayley-II or Bayley-III: what do the scores tell us? Dev Med Child Neurol, 2013. 55(11): p. 978-9.
11.Zhang, Z., et al., Associations of sleep characteristics with cognitive and gross motor development in toddlers. Sleep Health, 2022. 8(4): p. 350-355.
15.Dillhoff, A., et al. An automated assessment system for embodied cognition in children: from motion data to executive functioning. in In Proceedings of the 6th international Workshop on Sensor-based Activity Recognition and Interaction. 2019.
16.Ramesh Babu, A., et al. A multi-modal system to assess cognition in children from their physical movements. in In Proceedings of the 2020 International Conference on Multimodal Interaction. 2020.
17.Polsley, S., et al., Detecting Children’s Fine Motor Skill Development using Machine Learning. International Journal of Artificial Intelligence in Education, 2021(32): p. 991-1024.
18.Suzuki, S., Y. Amemiya, and M. Sato, Deep learning assessment of child gross-motor, in In 2020 13th International Conference on Human System Interaction (HSI). 2020, IEEE: Tokyo, Japan. p. 189-194.
24.Gerber, R.J., T. Wilks, and C. Erdie-Lalena, Developmental milestones: motor development. Pediatr Rev, 2010. 31(7): p. 267-76; quiz 277.

	27.Suggate, S., E. Pufke, and H. Stoeger, Do fine motor skills contribute to early reading development? Journal of Research in Reading, 2018. 41(1): p. 1-19.
**Additional Information:**	
**Question**	**Response**
Are you submitting this manuscript to a special series or article collection?	No
**Experimental design and statistics**<br><br>Full details of the experimental design and statistical methods used should be given in the Methods section, as detailed in our Minimum Standards Reporting Checklist. Information essential to interpreting the data presented should be made available in the figure legends.<br><br>Have you included all the information requested in your manuscript?	Yes
**Resources**<br><br>A description of all resources used, including antibodies, cell lines, animals and software tools, with enough information to allow them to be uniquely identified, should be included in the Methods section. Authors are strongly encouraged to cite Research Resource Identifiers (RRIDs) for antibodies, model organisms and tools, where possible.<br><br>Have you included the information requested as detailed in our Minimum Standards Reporting Checklist?	Yes
**Availability of data and materials**<br><br>All datasets and code on which the conclusions of the paper rely must be either included in your submission or deposited in publicly available repositories (where available and ethically appropriate), referencing such data using a unique identifier in the references and in the "Availability of Data and Materials"	Yes

Powered by Editorial Manager® and ProduXion Manager® from Aries Systems Corporation

section of your manuscript.<br><br>Have you have met the above requirement as detailed in our Minimum Standards Reporting Checklist?	

Manuscript

Click here to access/download;Manuscript;revised manuscript_SCE.docx

Multi-view child motor development dataset for AI-driven assessment of child development

Hye Hyeon Kim[1#], PhD; Jin Yong Kim[1#], BS; Bong Kyung Jang[1], BS; Joo Hyun Lee[1], BS; Jong Hyun Kim[1], BS; Dong Hoon Lee[1], BS; Hee Min Yang[1], BS;Young Jo Choi[1], BS;Myung Jun Sung[1], BS;Tae Jun Kang[2], BS; Eunah Kim[3], MA; Yang Seong Oh[3], MS; Jaehyun Lim[4], MD, PhD; Soon-Beom Hong[5, 6], MD, PhD; Kiok Ahn[7], PhD; Chan Lim Park[8], MS; Soon Myeong Kwon[8], BS; Yu Rang Park[1*], PhD

[1] Department of Biomedical Systems Informatics, Yonsei University College of Medicine, Seoul, Republic of Korea

[2] MISO Info Tech Co. Ltd., Seoul, Republic of Korea

[3] Maumdri Co. Ltd., Seoul, Republic of Korea

[4] Lumanlab, Inc., Seoul, Republic of Korea

[5] Division of Child and Adolescent Psychiatry, Department of Psychiatry, Seoul National University College of Medicine, Seoul, Republic of Korea

[6] Institute of Human Behavioral Medicine, Seoul National University Medical Research Center, Seoul, Republic of Korea

[7] GazziLabs, Inc., Seoul, Republic of Korea

[8] Smart Safety Laboratory Co. Ltd., Seoul, Republic of Korea

[#] HH Kim and JY Kim contributed equally to this paper

***Corresponding author:** Yu Rang Park, PhD

Department of Biomedical System Informatics, Yonsei University College of Medicine

50-1 Yonsei-ro, Seodaemun-gu, Seoul 03722, Korea

Phone: +82-2228-2493

Tel: 82-2-2228-2493, E-mail: yurangpark@yuhs.ac

ORCID iDs:

Hye Hyeon Kim [0000-0002-1928-4168]; Jin Yong Kim [0000-0002-2746-2710]; Joo Hyun Lee [0000-0002-3901-5846]; Jong Hyun Kim [0000-0002-4997-8398]; Yu Rang Park [0000-0002-4210-2094];

Abstract

Background: Children's motor development is a crucial tool for assessing developmental levels, identifying developmental disorders early, and taking appropriate action. Although the Korean Developmental Screening Test for Infants and Children (K-DST) can accurately assess childhood development, its dependence on parental surveys rather than reliable, professional observation limits it. This study constructed a dataset based on a skeleton of recordings of K-DST behaviors in children aged between 20 and 71 months, with and without developmental disorders. The dataset was validated using a child behavior artificial intelligence (AI) learning model to highlight its possibilities.

Results: The 339 participating children were divided into three groups by age. We collected videos of four behaviors by age group from three different angles and extracted skeletons from them. The raw data were used to annotate labels for each image, denoting whether each child performed the behavior properly. Behaviors were selected from the K-DST's gross motor section. The number of images collected differed by age group. The original dataset underwent additional processing to improve its quality. Finally, we confirmed that our dataset can be used in the AI model with 93.94%, 87.50%, and 96.31% test accuracy for the three age groups in an action recognition model. Additionally, the models trained with data including multiple views showed the best performance.

Conclusion: Ours is the first publicly available dataset that constitutes skeleton-based action recognition in young children according to the standardized criteria (K-DST). This dataset will enable the development of various models for developmental tests and screenings.

Keywords: skeleton-based action recognition; children motor development; AI model

1. Background

Motor development is essential for children's physical strength, movement, and identification of developmental difficulties. Motor development and control begin developing after birth and progress as children grow. Typically, children develop certain motor skills at a specific age; however, every child does not reach milestones at the same time [1]. Children with neurological problems, developmental delays, or disabilities may have difficulty with certain motor skills. Evaluating motor development can be a tool to assess a child's degree of development. Since a common clinical symptom of developmental milestones is not acquiring the developmental technology suitable for one's age, using simple evaluations to screen infants and toddlers with developmental problems early on [2]would be useful for planning appropriate treatment, rehabilitation, and education and improving prognoses. Additionally, early detection of developmental problems is crucial because delays can negatively affect a child's readiness to start school. Furthermore, it can cause issues with self-confidence because it is associated with the child's later achievements, such as literacy [3–5].

As a health examination project for infants and toddlers was implemented in South Korea in November 2007, the Korean Developmental Screening Test for Infants and Children (K-DST) [6] was developed to comprehensively determine the possibility of developmental disorders as

well as normal development. It evaluates children's behavior, including a wide age range for preschool infants under the age of six (4 months to 71 months), and deals with more comprehensive developmental areas. Although the K-DST was developed specifically for Korean children, it is used globally because it is based on international standards such as the National Health Screening Program for Infants and Children [7]. Among several developmental assessment tools such as the Ages and Stages Questionnaire[8, 9], Bayley Mental Development Index[10], Bayley Scales of Infant Development, Wechsler Preschool and Primary Scale of Intelligence, and Peabody Developmental Motor Scales[11], the K-DST was selected because it can be assessed without money and has age-specific behaviors to assess motor development. In addition, recent K-DST-based research has demonstrated through national cohorts that the K-DST is a robust assessment of child development [7].

Meanwhile, the majority of existing action recognition databases have been designed for adults. There have been many studies related to children's action cognition—such as an infant action database including 18 actions extracted from Instagram and YouTube [12], action recognition including seven actions in RGB for children aged 6–11 years[13], and skeleton driven action recognition including six actions for 32 children aged 6–9 years[14]—but there is no dataset that can be used publicly since they are all individual studies with minimal datasets or involve privacy issues.

Concerning the use of artificial intelligence, various studies have evaluated children's motor functions—evaluation of cognition with physical movements[15, 16], detection of machine learning-based fine motor skills[17], and evaluation of deep learning-based children's gross motor skills[18]—but they were all AI-based, model-oriented studies. Contrastingly, this study focused on presenting a dataset of children's gross motor skills for each age group.

This study developed a new dataset for motor development in young children, from toddlers to children, using the K-DST. Although multi-view recordings in previous studies[19-21] have attempted to enhance the explanatory power with more data from the combinations of anatomical feature locations from various angles, this method was selected for the following three additional reasons: 1) to consider the characteristics of children who are free to move and are not easy to control, 2) to confirm the assumption that there may be a specific angle that captures a specific behavior well, and 3) to confirm the assumption that the combination of data from certain angles can improve data learning performance results. This dataset can be used as an essential resource for the development of artificial intelligence algorithms to determine children's behavior and evaluate their development.

2. Methods

2.1 Participants

All experiments were performed in accordance with the ethical principles of the Declaration of Helsinki. This study was approved by the Institutional Review Board of Severance Hospital, Yonsei University College of Medicine, and the requirement for informed consent was waived (Institutional Review Board [IRB]number:4-2021-0845). All caregivers provided written informed consent for data collection and subsequent analyses. All efforts were made to minimize the children's discomfort. The participants were children aged between 20 and 71 months from all over the country and were recruited from daycare centers, kindergartens, primary hospitals (pediatrics and adolescent medicine), and Internet communities. They were divided into three age groups: 20–35 months (Group A), 36–53 months (Group B), and 54–71

months (Group C). The total participants included 399 children, with a sex ratio of 53:47 (Male: Female). Table 1 provides detailed information and sex ratios of the participants.

Table 1 Distribution of participants by age groups.

	Total (n=399)	Group A (20–35 months, n=136)	Group B (36–53 months, n=106)	Group C (54–71 months, n=157)
Sex (n, %)				
Male	213 (53%)	68 (50%)	57 (54%)	88 (56%)
Female	186 (47%)	68 (50%)	49 (46%)	69 (44%)

2.2 Type of behavior

Our dataset was collected based on the K-DST—a tool created for the accurate examination of developmental delays[22] and health management of infants and children by reflecting the characteristics of Korean infants and children. It is intended for infants and children between 4 and 71 months and includes 48 items for each age group.

Among these 48 items, core tasks were selected for each age group through consultations with three pediatricians and 15 child development experts based on the literature review, such as previous motor development guidelines[23, 24]. The principal criteria for selecting core tasks were: 1) developmental milestones, 2) physical and cognitive abilities, and 3) behaviors that measure various motor skills of each age group. First, developmental milestones were identified based on a 2010 study published in Pediatric in Review[24]. Second, age-appropriate physical and cognitive abilities were considered. Simple tasks were selected for younger children with limited coordination, while coordination-based tasks were adopted for older children. Third, various gross motor functions were evaluated by examining the total muscle function through various movements involving the whole body, upper body, or lower body.

The representative motor development behaviors for each age group were selected to evaluate children's gross motor skills at that age. Twelve motor development tasks were defined, with four tasks representing each age group (Table 2).

Based on the literature review, 18 pediatricians and experts discussed representative behaviors for each age group and selected specific actions as measurements of behavioral development.

Table 2 Four core motor development tasks for the three age groups based on the K-DST.

Group	ID	Action Description
Group A	1-1	Place his/her feet together and climb up the stairs one by one without holding onto the railing.
	1-2	Place his/her feet together and go down the stairs one by one without holding onto anything.
	1-3	Raise his/her arms and throw the ball over his/her head while standing.
	1-4	Stand on one foot for a second without holding onto anything.
Group B	2-1	Stand on one foot for more than three seconds without holding onto anything.
	2-2	Hop 2–3 steps on one foot.
	2-3	Put his/her feet together and make a big jump.
	2-4	Receive a big ball using both his/her arms and chest.
Group C	3-1	Stop a rolling ball with his/her feet.
	3-2	Bounce a ball on the floor once.
	3-3	Jump over a rope tied high below his/her knees.
	3-4	Jump rope once.

2.3 Experimental setup and data acquisition

Participants were asked to perform four behaviors at least five to ten times, and the behaviors were video recorded using RGB cameras. The number of trials for each behavior depended on the child's condition and cooperation. Each behavior was recorded simultaneously using three cameras (Figure 1A). The distance and angle of the cameras depended on the child's age group, the details of which are described in Figure 1B. All videos were recorded using a SONY DSC-RX100 with a resolution of 1920x1080 at 30 fps. Figure 1C shows a portion of the videos

recorded from three angles for Behavior 1 of Child B010. It represents a snapshot of a Group B child's video for Behavior 1 (standing on one foot for more than three seconds without holding onto anything): view 1 (front), view 2 (right), and view 3 (left). To measure the behavior of all children, the distance from the camera for each age group was defined differently based on the child with the maximum height in each age group.

2.4 Annotation of behavior

At the labeling stage, the criteria for evaluating child development were determined based on the opinions of 12 pediatricians and child development experts (Table 3). Two evaluation processes were conducted based on these developmental evaluation criteria. In the first stage, 15 child development experts with board-certified behavior analyst certificates or equivalent experience conducted an evaluation. At this stage, behaviors were divided into 0 (Bad), 1 (Good), and 2 (Perfect), according to each child's performance of the behavior. This evaluation method utilized a 3-point scale, which is a modification of the 4-point scale used in the K-DST. The former regards 0 (not able to do at all) and 1 (not able to do it) in the 4-point scale as one score (0), 2 (able to do it) as 1, and 3 (can do it well) as 2. Two or more experts simultaneously evaluated each child's behavior to increase the reliability of the evaluation results. In the second stage, pediatricians conducted an overall review based on the evaluation results of the first stage. If the results of the first evaluation stage for child behavior assessment did not match, a consensus was reached through discussion between experts, and the first evaluation was conducted again. The evaluation was conducted in two stages for three reasons. First, the opinions of pediatricians and child development experts were considered. Second, the assessments were double-checked to increase their accuracy. Third, the pediatricians' role in the final stage was more confirmatory.

Table 3 Labeling criteria for child behavior.

Group	Behavior ID	Labeling criteria
Group A	A01<br>Go up the stairs	0 (bad): He/She cannot climb up the stairs.<br>1 (good): He/She can climb up the stairs but pauses a little.<br>2 (perfect): He/She can climb up the stairs without difficulty.
	A02<br>Go down the stairs	0 (bad): He/She cannot go down the stairs.<br>1 (good): He/She can go down the stairs but pauses a little.<br>2 (perfect): He/She can go down the stairs without difficulty.
	A03<br>Throw the ball	0 (bad): He/She cannot throw the ball over his/her head.<br>1 (good): He/She can throw the ball over his/her head but staggers.<br>2 (perfect): He/She can throw the ball over his/her head while standing straight.
	A04<br>Stand on one foot	0 (bad): He/She cannot stand on one foot even for a moment.<br>1 (good): He/She can stand on one foot for a second but staggers.<br>2 (perfect): He/She can stand on one foot for a second without staggering.
Group B	B01<br>Stand on one foot	0 (bad): He/She cannot stand on one foot even for a moment.<br>1 (good): He/She can stand on one foot for more than three seconds but staggers.<br>2 (perfect): He/She can stand on one foot for more than three seconds without staggering.
	B02<br>Hop 2–3 steps	0 (bad): He/She cannot hop even once.<br>1 (good): He/She can hop 2–3 steps but pauses a little.<br>2 (perfect): He/She can hop 2–3 steps without difficulty.
	B03<br>Long jump	0 (bad): He/She cannot jump with his/her feet together.<br>1 (good): He/She can jump with his/her feet together.<br>2 (perfect): He/She can jump a long distance with his/her feet together.
	B04<br>Receive the ball	0 (bad): He/She cannot receive a ball.<br>1 (good): He/She can receive a ball using both arms and chest but staggers after receiving it.<br>2 (perfect): He/She can receive a ball using both arms and chest without staggering.
Group C	C01<br>Stop the rolling ball	0 (bad): He/She cannot stop a rolling ball with his/her foot.<br>1 (good): He/She can stop a rolling ball with his/her foot.<br>2 (perfect): He/She can stop a rolling ball with his/her sole.
	C02<br>Bounce the ball	0 (bad): He/She cannot bounce the ball on the floor at all.<br>1 (good): He/She can bounce the ball on the floor once.<br>2 (perfect): He/She can bounce the ball on the floor once stably.

C03<br>Jump over the rope	0 (bad): He/She cannot jump over the rope tied below his/her knee level.<br>1 (good): He/She can jump over the rope with a little hesitation.<br>2 (perfect): He/She can jump over the rope without hesitation.
C04<br>Jump rope	0 (bad): He/She cannot jump rope even once.<br>1 (good): He/She can jump rope with a little hesitation.<br>2 (perfect): He/She can jump rope without hesitation.

2.5 Preprocessing of children's behavior

The OpenPose algorithm [25] was used to obtain human skeletal data from the RGB videos. OpenPose is a pose-estimation algorithm that extracts joint coordinates from RGB videos in three channels (x coordinates, y coordinates, and confidence scores). The BODY_25 format (Figure 2) was used to obtain 25 joint coordinates per frame. Additional post-processing was performed on the raw skeletons. First, there were missing joints in the outputs from the OpenPose algorithm; therefore, the neck joint was set as the core joint, and skeletons missing this core joint were removed because they were unreliable.

Second, although OpenPose detects multiple people in a single frame, the *n-th* person at frame *t* and the *n-th* person at time *t-1* may not be the same because they are simply listed without object identification. To solve this problem, skeletons were aligned based on the core joint (neck)[14]. Assuming that there is a neck coordinate for person 1 in frame *t*, the Euclidean distance from the neck coordinates of all people in the previous frame is calculated and connected to the closest person. Finally, the original coordinates were converted to represent the relative position based on the core joint and scaled to obtain values between 0.5 and 0.5, which can be calculated as

$$x = (x/frame\_width) - x_{neck}$$

$$y = (y/frame\_height) - y_{neck}$$

2.6 Evaluation for action recognition

The dataset was evaluated by training the deep learning model MS-G3D, a GCN-based action recognition model[26]. Since only well-performed actions should be used as input data for action recognition, only data that received a score of one or two were used. The models were trained by age group, and combinations of camera views (three angles: front, left, and right) were explored by training with data from specific views. Therefore, there were 21 models: three age groups and seven view combination settings for each age group. Each model was trained with data including specific views depending on its view combination setting. Interconnections of multi-views were not considered in the models. Models were trained with the data from each view independently.

The mean length of data in age groups A, B, and C were 136, 167, and 87 frames, respectively. Videos were normally shorter than 300 frames based on the review of the video length histogram (see Supplement Figure S3). Therefore, the maximum length of input was set as 300 frames because the GCN-based action recognition models only accept inputs of the same length as RNNs. It was padded by zero if the sample length was shorter than 300 frames and sliced to 300 frames if the sample length was longer than 300 frames.

Initially, we trained for 100 epochs to optimize the number of epochs for training. Since the models converged before 50 epochs, we trained for 50 epochs in the entire experiment (see Supplement Figure S2). We used an SGD optimizer with a weight decay of 0.001, a base learning rate of 0.01(a high base learning rate is common in GCN-based action recognition model training), and a MultiStepLR learning rate scheduler with milestones (20, 30, 40),

gamma 0.1. This hyperparameter setting was fixed across all models to only evaluate the effect of the combination. Additionally, the whole random seed was fixed to 100.

The dataset was split into three subsets based on the participants while considering the overfitting problem (see Supplement S1): training (80%), validation (10%), and testing (10%). In age group A, the training, validation, and testing sets included 4,368 samples of 104 participants, 579 samples of 14 participants, and 593 samples of 13 participants, respectively. In age group B, the training, validation, and testing sets included 2,685 samples of 80 participants, 309 samples of 11 participants, and 360 samples of 8 participants, respectively. In age group C, the training, validation, and testing sets included 5049 samples of 125 participants, 687 samples of 17 participants, and 597 samples of 14 participants, respectively. There were seven combinations of camera views, and each setting had the same behavior data from the same children in the training, validation, and testing sets.

3. Results

3.1 Data distribution

The data distribution of the dataset is presented in Table 4. Except for a few actions, the overall distribution was unbalanced. The distribution was most unbalanced in Group C, the oldest group with the largest ratio of perfect actions. The sex ratios for each behavior were balanced in all age groups.

Table 4 Data distribution of the child behavior dataset.

Group	Behavior ID	Number of participants (female%)	Number of videos for each label			Total
			Bad (n, %)	Good (n, %)	Perfect (n, %)	
Group A	A01	136 (50%)	372	303	1130	1805

(n=136)	Climb up the stairs		(20%)	(17%)	(63%)	
	A02	136 (50%)	400	300	1091	1791
	Go down the stairs		(22%)	(17%)	(61%)	
	A03	135 (50%)	249	445	1119	1813
	Throw the ball		(14%)	(25%)	(61%)	
	A04	136 (50%)	620	627	543	1790
	Stand on one foot		(35%)	(35%)	(30%)	
Group B	B01	98 (47%)	182	504	519	1205
(n=106)	Stand on one foot		(15%)	(42%)	(43%)	
	B02	96 (47%)	471	270	315	1056
	Hop 2–3 steps		(45%)	(25%)	(30%)	
	B03	103 (46%)	180	213	705	1098
	Long jump		(16%)	(19%)	(64%)	
	B04	103 (48%)	468	348	486	1302
	Receive the ball		(36%)	(27%)	(37%)	
Group C	C01	154 (45%)	278	415	1315	2008
(n=157)	Stop the rolling ball		(14%)	(21%)	(65%)	
	C02	152 (45%)	204	294	1471	1969
	Bounce the ball		(10%)	(15%)	(75%)	
	C03	148 (47%)	108	264	1648	2020
	Jump over the rope		(5%)	(13%)	(82%)	
	C04	137 (47%)	767	534	408	1709
	Jump rope		(45%)	(31%)	(24%)	

3.2 Action recognition

We explored combinations of camera views by training with data from specific views to determine the most informative one. In Group A, the combination of all views showed the best performance (93.94%). In Group B, the front-right combination showed the best performance (88.33%). In Group C, the combination of all views showed the best performance (96.31%) (Table 5). This result indicates that if the number of views is higher, the performance is better because most of the upper ranks are combinations of multiple views. However, in the results using only a single view, the front view showed consistently good performance. This result suggests that the front view was the most informative. Therefore, in case of scarce resources, training the deep learning model using data from the front view is sufficient.

Additionally, the confusion matrices of three-view models and single-view models were obtained (see Supplement Figure S4). The confusion matrices show that the diagonal of the

three-view model's matrix had higher values than the front-view model's matrix. In other words, three-view models showed better performance than single-view models.

Table 5 Classification accuracy comparison by camera view combination.

No.	Camera view combination	Top-1 (%)		
		Group A	Group B	Group C
1	Front, Left, Right	93.94	87.50	96.31
2	Front, Right	93.69	88.33	94.22
3	Front, Left	92.17	85.83	95.23
4	Right, Left	91.67	85.83	96.23
5	Front	92.93	82.50	90.95
6	Right	92.93	77.50	92.96
7	Left	85.35	85.83	91.46

Conclusion

The dataset presented in this study consisted of young children divided into three age groups, based on the K-DST, performing four representative behaviors for each age group that were recorded and collected from three different angles. To represent child development datasets, we first defined core behaviors in the child development process by age group through active discussions with a group of child development experts, including pediatricians. Second, we established evaluation criteria in three stages for each behavior for clear and reliable evaluation. The data were collected from 399 children. Each video of child behavior was manually labeled using a 3-point scale for the evaluation of motor development created by 15 developmental assessment experts and three pediatricians. As a result of applying a deep learning-based action recognition model to verify the quality of the developed dataset, data collected from two or more directions performed better than individual directions. Our dataset is the first publicly

accessible dataset that enables the identification and evaluation of young children's actions and motor development based on their skeletons. This study emphasized gross motor skills based on a previous study[7] that found the gross motor to have more accuracy than the fine motor in the K-DST for children's motor skill evaluation. However, other previous studies[3, 27] have shown that fine motor skills are also valuable in evaluating children's motor skills. In our future work, we will compare fine and gross motor skill evaluations to enhance the accuracy of child development evaluation. Additionally, the dataset will be extended to include children with and without developmental disabilities. It can be utilized to develop early diagnostic prediction models using AI techniques such as machine learning. Since the dataset provided in this study includes scores, it can be used to develop a model for predicting scores. Furthermore, it can be utilized as the basis for developing screening tools for children's quantitative motor development levels (body maturity). Moreover, it was found that utilizing the multi-view data had positive effects on the model training. In our future work, we will measure the effect of multiple views by combining multiple data as an extended concept of multiple data utilization.

Data Availability

All collected data is available in our Github repository [28]. All supporting data and materials are also available in the *GigaScience* GigaDB database [29].

Availability of source code and requirements

Project name: **Multi-view child motor development dataset for AI-driven assessment of child development**

-
- Project home page: https://github.com/DigitalHealthcareLab/22ActionRecognitionTool.
- Operating system(s): Linux

- Programming language: Python3
- Other requirements: PyTorch>=1.2.0, pyyaml, tensorboardX, tqdm, glob
- License: MIT license
- RRID: SCR_023552

List of abbreviations

K-DST, Korean Developmental Screening Test for Infants and Children; GCN, Graph Convolutional Networks; RGB, red-green-blue; IRB, Institutional Review Board

Competing interests

The authors declare that they have no competing interests.

Funding

This research was supported by a grant for the R&D project, funded by the National Center for Mental Health (grant number: MHER22A01).

This paper used datasets from machine learning data collection projects funded by the Ministry of Science & ICT and the National Information Society Agency (NIA, S. Korea).

Authors' contributions

Conceptualization: HH Kim, JY Kim, and YR Park; Methodology: HH Kim, JY Kim, and YR Park; Data Collection: E Kim, YS Oh, JH Kim, and SB Hong; Data Cropping and Parsing: BK Jang, JH Lee, JH Kim, DH Lee, HM Yang, YJ Choi, MJ Sung; Data Curation: KA Ahn; Data Evaluation: CL Park and SM Kwon; Annotation Validation: E Kim, YS Oh, JH Kim, and SB Hong; Writing: HH Kim, JY Kim, and YR Park; AI Modeling and Validation: JY Kim;

Supervision: YR Park; Project Administration: TJ Kang. All authors have read and agreed to the published version of the manuscript.

References

1. Group, W.H.O.M.G.R.S., *WHO Motor Development Study: windows of achievement for six gross motor development milestones.* Acta Paediatr Suppl, 2006. **450**: p. 86-95.
2. Karmel, B.Z., et al., *Early medical and behavioral characteristics of NICU infants later classified with ASD.* Pediatrics, 2010. **126**(3): p. 457-67.
3. Grissmer, D., et al., *Fine motor skills and early comprehension of the world: two new school readiness indicators.* Dev Psychol, 2010. **46**(5): p. 1008-17.
4. Kamphorst, E., et al., *Emerging School Readiness Profiles: Motor Skills Matter for Cognitive- and Non-cognitive First Grade School Outcomes.* Front Psychol, 2021. **12**: p. 759480.
5. Duncan, G.J., et al., *School readiness and later achievement.* Dev Psychol, 2007. **43**(6): p. 1428-1446.
6. Jang, C.H., et al., *Clinical Usefulness of the Korean Developmental Screening Test (K-DST) for Developmental Delays.* Ann Rehabil Med, 2019. **43**(4): p. 490-496.
7. Chung, H.J., et al., *Development of the Korean Developmental Screening Test for Infants and Children (K-DST).* Clin Exp Pediatr, 2020. **63**(11): p. 438-446.
8. Romero Otalvaro, A.M., et al., *ASQ-3: Validation of the Ages and Stages Questionnaire for the detection of neurodevelopmental disorders in Argentine children.* Arch Argent Pediatr, 2018. **116**(1): p. 7-13.
9. Veldman, S.L., et al., *Prevalence and risk factors of gross motor delay in pre-schoolers.* J Paediatr Child Health, 2020. **56**(4): p. 571-576.
10. Bos, A.F., *Bayley-II or Bayley-III: what do the scores tell us?* Dev Med Child Neurol, 2013. **55**(11): p. 978-9.
11. Zhang, Z., et al., *Associations of sleep characteristics with cognitive and gross motor development in toddlers.* Sleep Health, 2022. **8**(4): p. 350-355.
12. Balasathiya, S.S., S.M.M. Roomi, and B. Sathyabama. *Infant Action Database: A Benchmark for Infant Action Recognition in Uncontrolled condition*. in *Journal of Physics: Conference Series*. 2021. IOP Publishing. doi: 10.1088/1742-6596/1917/1/012019
13. Turarova, A., et al., *Child Action Recognition in RGB and RGB-D Data.* Hri'20: Companion of the 2020 Acm/Ieee International Conference on Human-Robot Interaction, 2020: p. 491-492.
14. Silva, V., et al., *Skeleton Driven Action Recognition Using an Image-Based Spatial-Temporal Representation and Convolution Neural Network.* Sensors (Basel), 2021. **21**(13).
15. Dillhoff, A., et al. *An automated assessment system for embodied cognition in children: from motion data to executive functioning*. in *In Proceedings of the 6th international Workshop on Sensor-based Activity Recognition and Interaction*. 2019.
16. Ramesh Babu, A., et al. *A multi-modal system to assess cognition in children from their physical movements*. in *In Proceedings of the 2020 International Conference on Multimodal Interaction*. 2020. https://doi.org/10.1145/3382507.3418829

17. Polsley, S., et al., *Detecting Children's Fine Motor Skill Development using Machine Learning.* International Journal of Artificial Intelligence in Education, 2021(32): p. 991-1024.
18. Suzuki, S., Y. Amemiya, and M. Sato, *Deep learning assessment of child gross-motor,* in *In 2020 13th International Conference on Human System Interaction (HSI)*. 2020, IEEE: Tokyo, Japan. p. 189-194.
19. Nicora, E., et al., *The MoCA dataset, kinematic and multi-view visual streams of fine-grained cooking actions.* Sci Data, 2020. **7**(1): p. 432.
20. Tang, J., et al., *Improved multi-view privileged support vector machine.* Neural Netw, 2018. **106**: p. 96-109.
21. Wang, Y., et al., *Discriminative Multi-View Dynamic Image Fusion for Cross-View 3-D Action Recognition.* IEEE Trans Neural Netw Learn Syst, 2021. **PP**.
22. Shin, S.M., *Should we regularly evaluate the neurodevelopmental status of moderate and late preterm infants?* Clin Exp Pediatr, 2020. **63**(6): p. 217-218.
23. McWilliams, C., et al., *Best-practice guidelines for physical activity at child care.* Pediatrics, 2009. **124**(6): p. 1650-9.
24. Gerber, R.J., T. Wilks, and C. Erdie-Lalena, *Developmental milestones: motor development.* Pediatr Rev, 2010. **31**(7): p. 267-76; quiz 277.
25. Cao, Z., et al., *OpenPose: Realtime Multi-Person 2D Pose Estimation Using Part Affinity Fields.* IEEE Trans Pattern Anal Mach Intell, 2021. **43**(1): p. 172-186.
26. Liu, Z., et al. *Disentangling and unifying graph convolutions for skeleton-based action recognition*. in *Proceedings of the IEEE/CVF conference on computer vision and pattern recognition*. 2020. https://openaccess.thecvf.com/content_CVPR_2020/papers/Liu_Disentangling_and_Unifying_Graph_Convolutions_for_Skeleton-Based_Action_Recognition_CVPR_2020_paper.pdf
27. Suggate, S., E. Pufke, and H. Stoeger, *Do fine motor skills contribute to early reading development?* Journal of Research in Reading, 2018. **41**(1): p. 1-19.
28. DigitalHealthcareLab. 22 Action Recogntion Tool GitHub https://github.com/DigitalHealthcareLab/22ActionRecognitionTool
29. Kim HH; Kim JY; Jang BK; Lee JH; Kim JH; Lee DH; Yang HM; Choi YJ; Sung MJ; Kang TJ; Kim E; Oh YS; Lim J; Hong S; Ahn K; Park CL; Kwon SM; Park YR. Supporting data for "Multi-view child motor development dataset for AI-driven assessment of child development" GigaScience Database 2023. http://dx.doi.org/10.5524/102382

Figure Titles

Figure 1 Experimental setup and data acquisition for video-based child behavior data. (A) Setting up an environment for documenting child behavior. (B) Camera angle and distance between child and camera according to age group. (C) Snapshot examples of a child's

behavior video for Group B Behavior 1 (stand on one foot for more than three seconds without holding onto anything): view 1 (front), view 2 (right), and view 3 (left).

Figure 2. Snapshot examples of the skeleton videos extracted from the same videos in Figure 1C. BODY_25 format was used with an output of 25 joints. These snapshots are for illustrative purposes only, and the actual data is the list of joint coordinates from entire frames. This list contains all captured joint coordinates from one video.

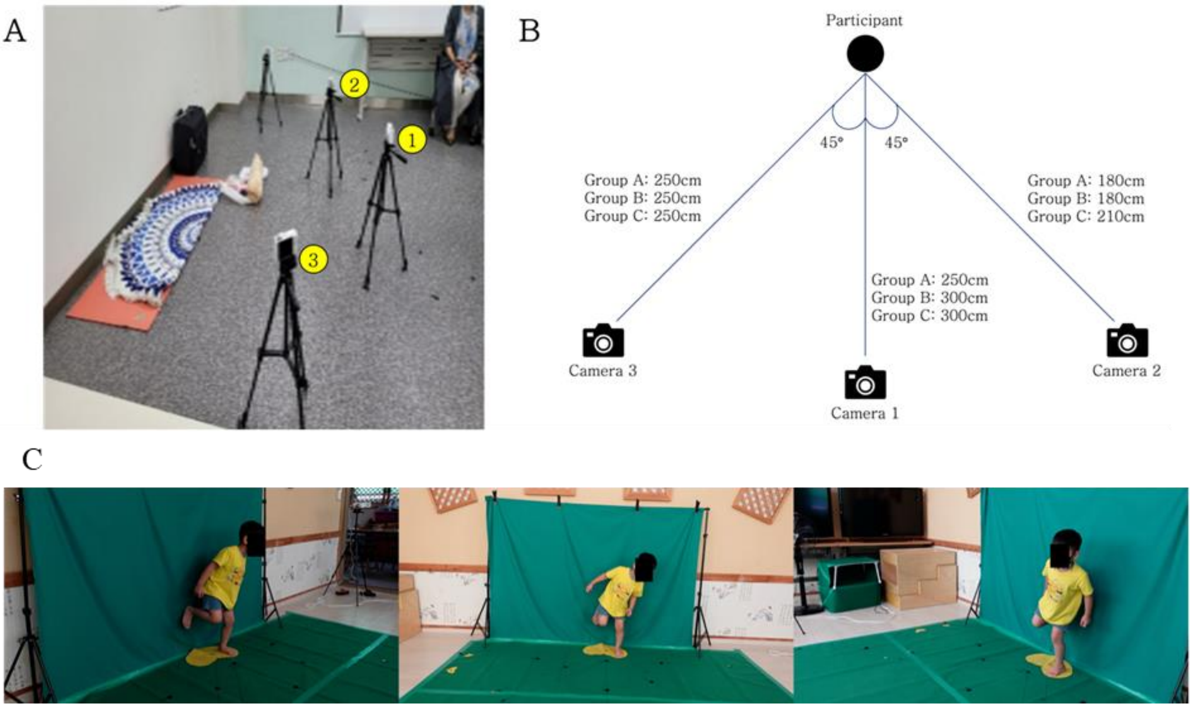
A
B
C
Participant
45°
45°
Group A: 250cm
Group B: 250cm
Group C: 250cm
Group A: 180cm
Group B: 180cm
Group C: 210cm
Group A: 250cm
Group B: 300cm
Group C: 300cm
Camera 3
Camera 1
Camera 2

Figure

Click here to access/download;Figure;Figure_2.docx

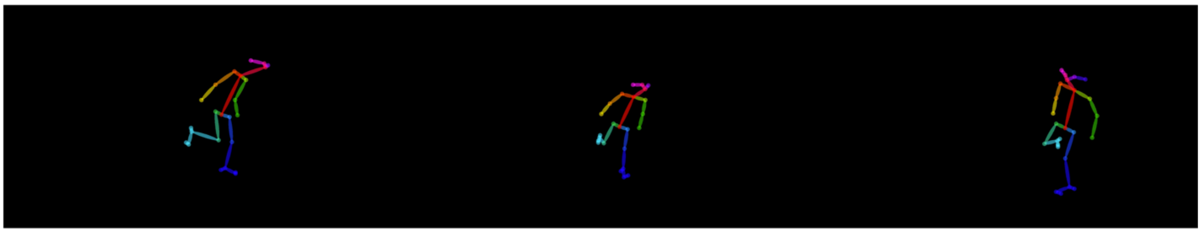

Click here to access/download
Supplementary Material
GigaDBUploadForm_Final.xlsx

Click here to access/download
Supplementary Material
Supplemental material.docx
