# Supplementary material for: Multiview child motor development dataset for AI-driven assessment of child development
Source: Gigascience. 2023 May 27;12:giad039. doi: 10.1093/gigascience/giad039 (PMC10220505; doi:10.1093/gigascience/giad039)

**Supplemental material**

**Figure S1. Loss graphs of each model.**

The yellow lines display the valid loss, while the blue lines show the training loss. Our experiment consisted of 21 models, encompassing three age groups and seven view settings per age. Each column presents graphs for a particular age group, ordered as follows: Age Group A, Age Group B, Age Group C. Each row displays graphs for a specific view setting, arranged as follows: View123, View12, View13, View23, View1, View2, View3. Across all settings, the training loss and valid loss converged simultaneously, and none of the 21 models exhibited any signs of loss explosion, indicating the absence of overfitting during the model training process."


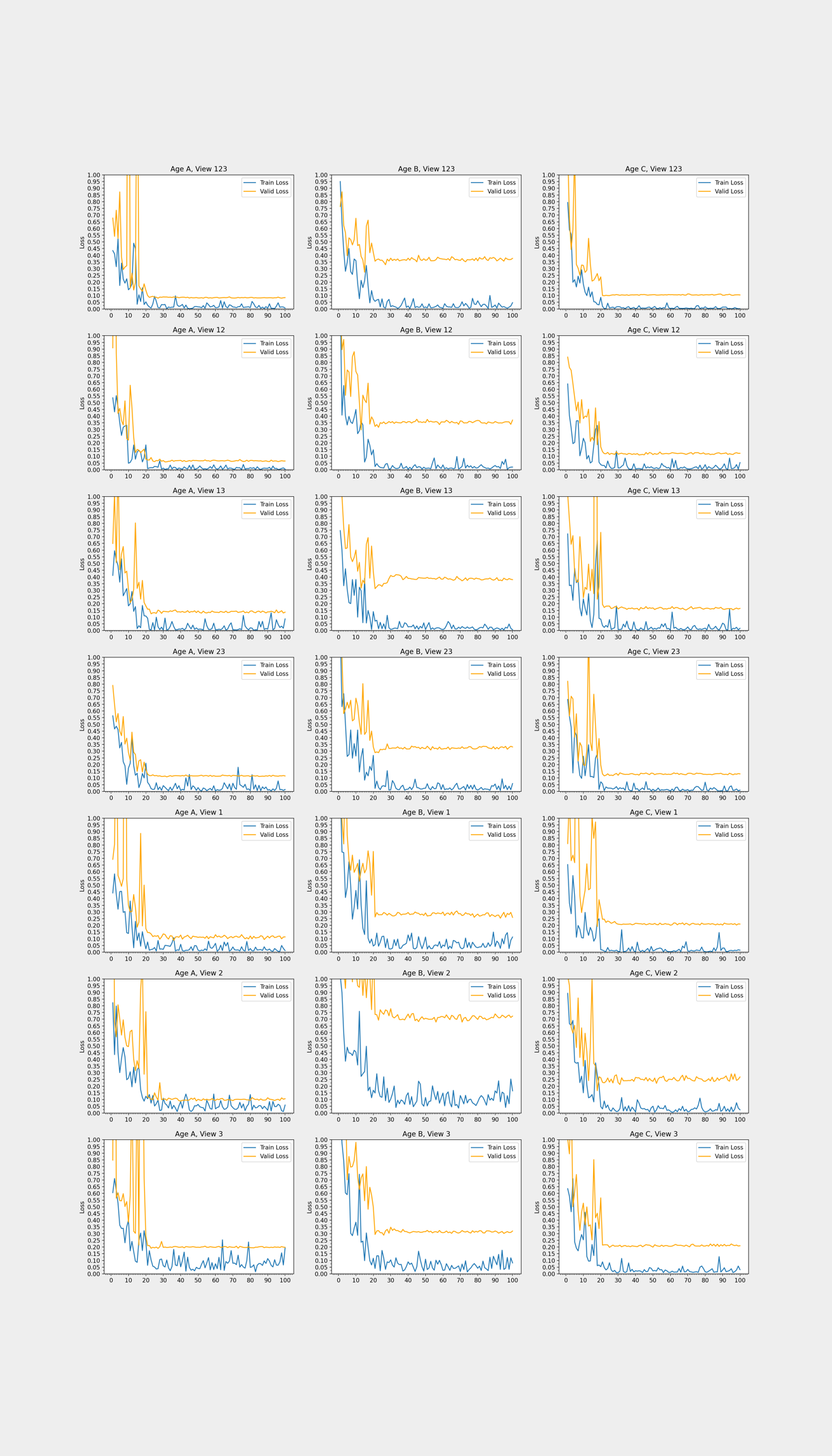


**Figure S2.** **Training graphs of each model.**

The yellow lines indicate train loss, and the blue lines represent train accuracy. Our experiment consisted of a total of 21 models, comprising 3 age groups and 7 view settings per age group. Each column in the graphs corresponds to one age group, ordered as follows: age group A, age group B, and age group C. Furthermore, each row displays the graphs for one view combination, following the sequence of View123, View12, View13, View23, View1, View2, and View3. The graphs exhibit train accuracy and train loss for 100 epochs, revealing that the models converged before 50 epochs of training, despite the absence of pre-training.


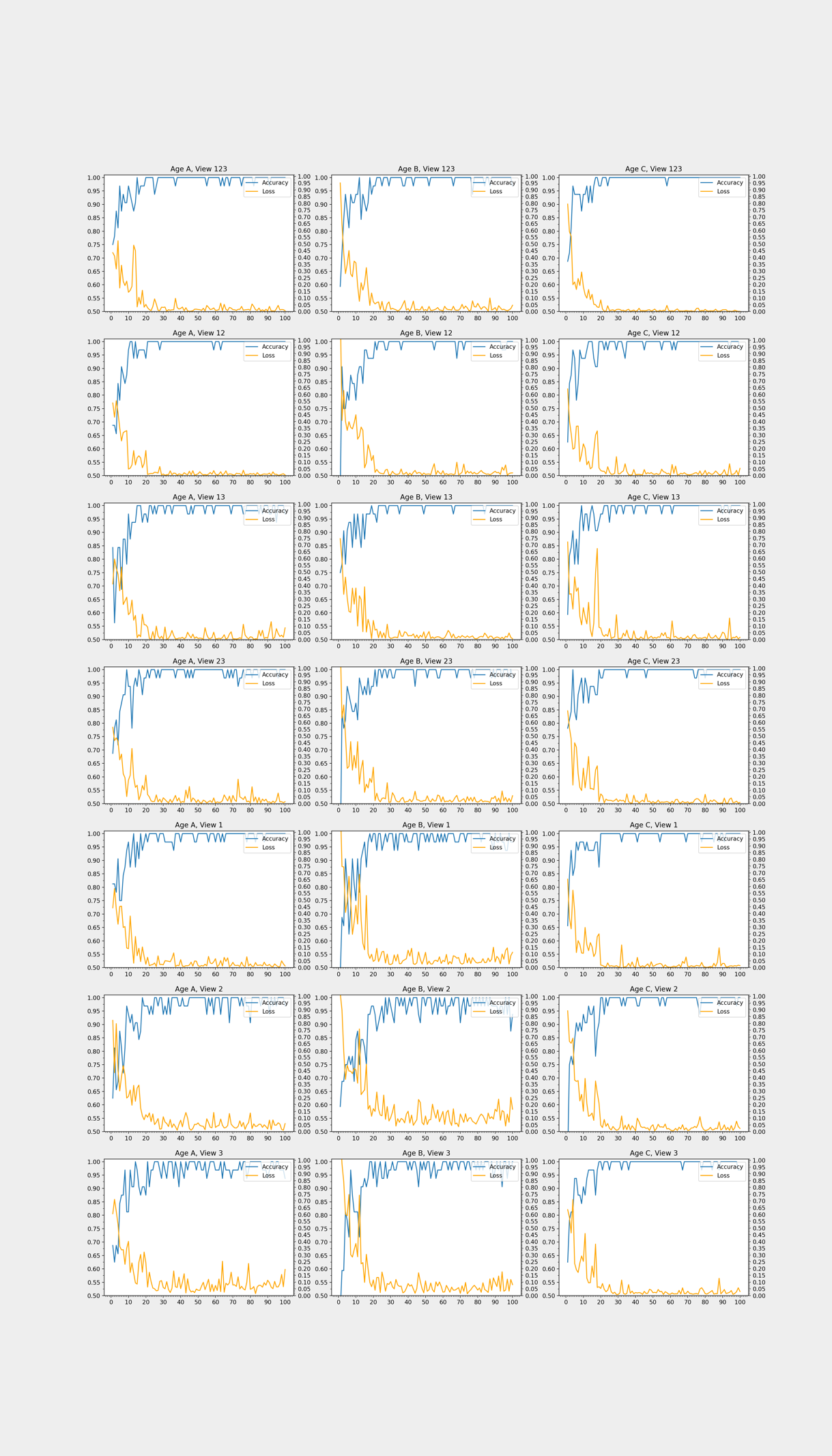


**Figure S3.** **Video frame length histograms for each age group.**

The x-axis represents the frame length of each video, while the y-axis indicates the number of videos. The histograms indicate that most videos were less than 300 frames in length; consequently, we designated the maximum frame length for model inputs as 300 frames.


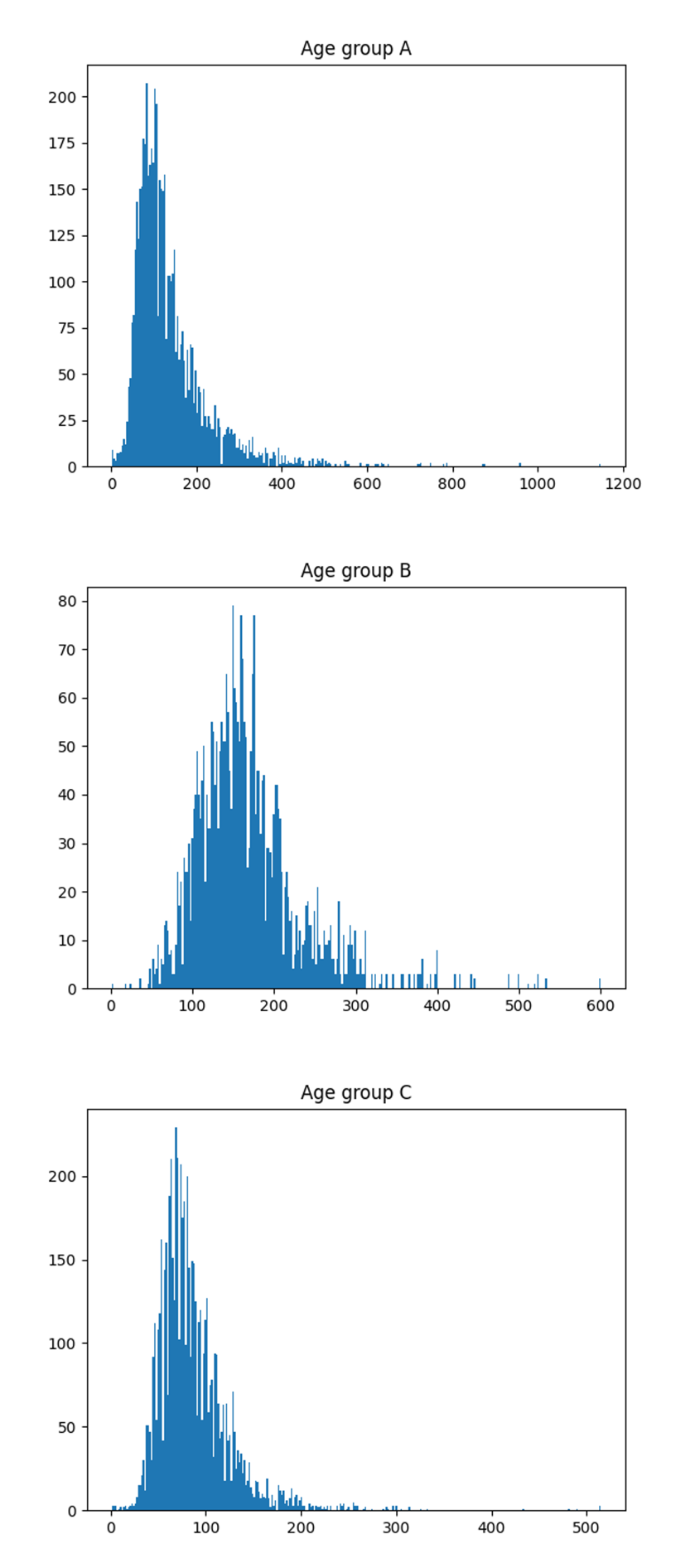


**Figure S4.** **Confusion matrices of each age group model predictions for testing set.**

(A), (B), and (C) on the left column are for single (front) view models, while (D), (E), and (F) on the right column are for three-view models. The confusion matrices indicate that models trained with three-view data outperformed those trained with front-view data. Specifically, the diagonal values of the three-view model's matrix were higher compared to those of the front-view model, indicating superior performance of the three-view models over the single-view models.


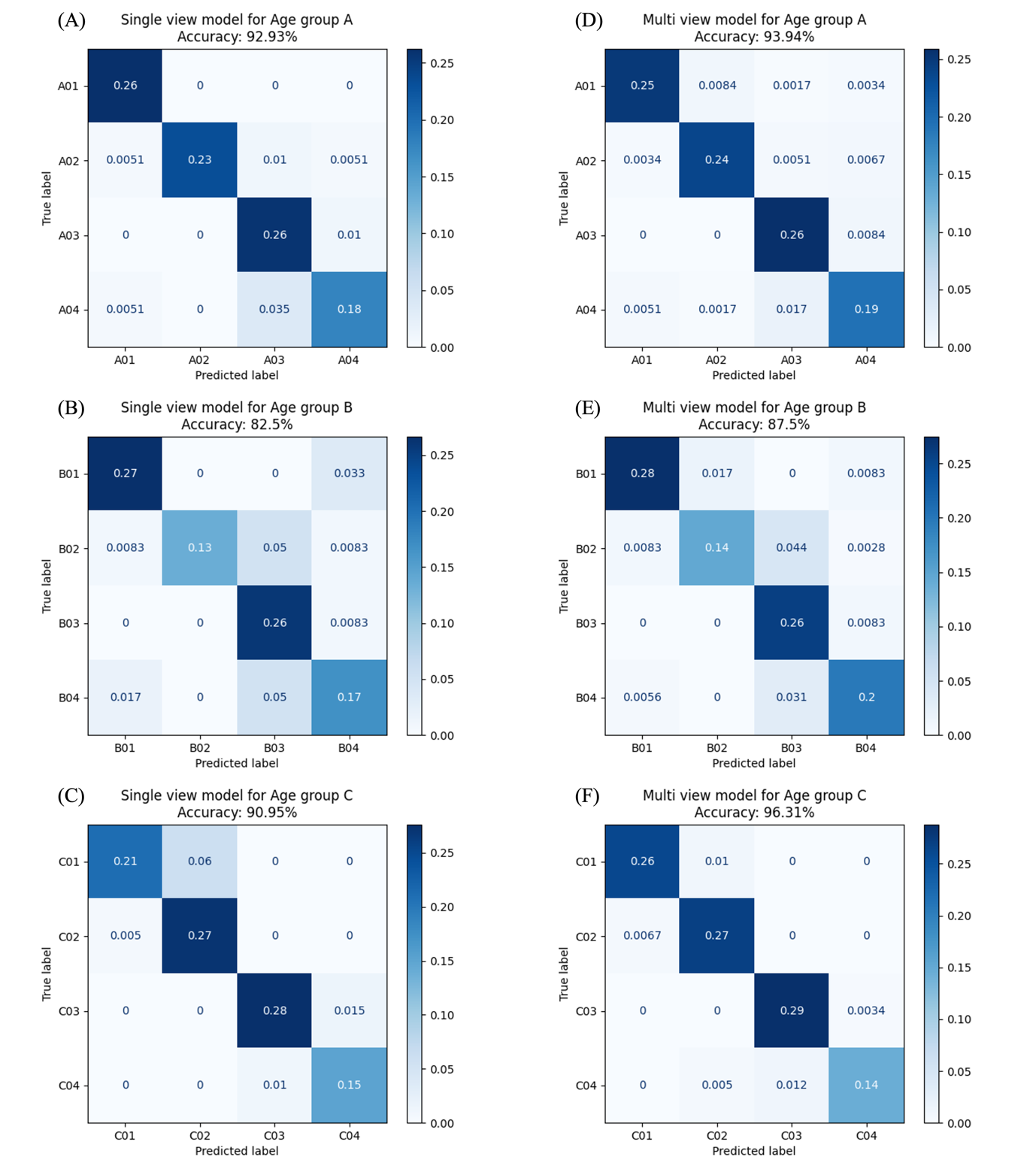


**Figure S5 Training graphs of combination model in View123 setting for age group A.**

((A) represents the train and validation accuracy graph, while (B) depicts the train and validation loss graph. The blue lines indicate the train accuracy and loss, and the yellow lines indicate the validation accuracy and loss. The graphs illustrate that the model failed to converge after 100 epochs of training when utilizing a data combination approach that incorporated features from multiple views. Furthermore, the validation accuracy steadily decreased after the 30th epoch, and the validation loss increased after the 30th epoch, indicative of overfitting issues. Thus, the decision was made to independently train the model using data from each view."


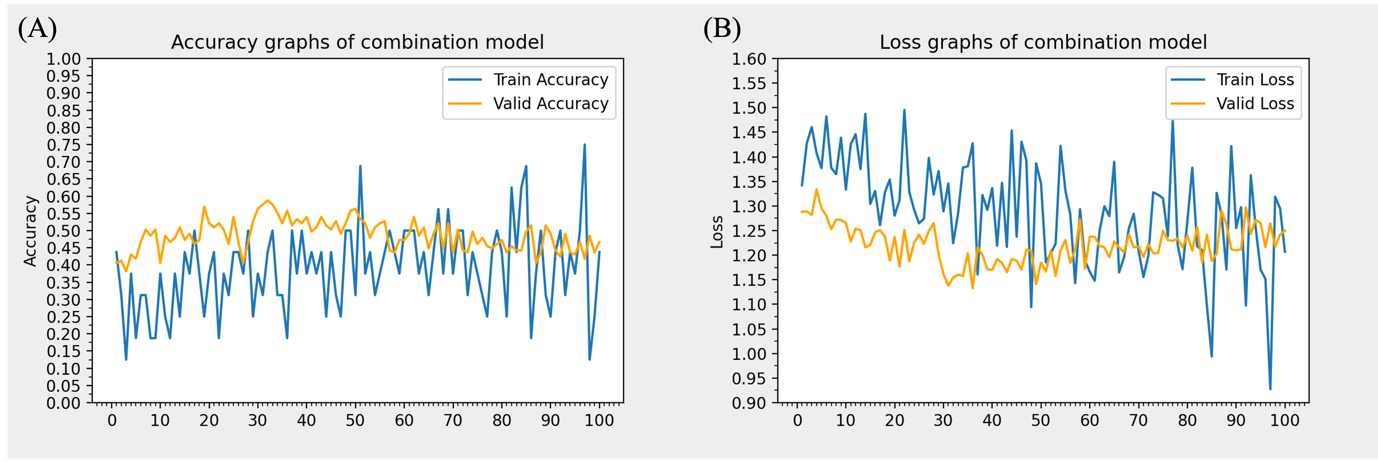

Supplement: giad039_Supplemental_File [file giad039_supplemental_file.docx]
