# Supplementary material for: Multiview child motor development dataset for AI-driven assessment of child development
Source: Gigascience. 2023 May 27;12:giad039. doi: 10.1093/gigascience/giad039 (PMC10220505; doi:10.1093/gigascience/giad039)
Supplement: giad039_Response_to_Reviewer_Comments_Original_Submission [file giad039_response_to_reviewer_comments_original_submission.pdf]

Author's Response To Reviewer Comments

Close

Rebuttal Letter

March 15, 2023
Travis Sanchez,
Editors-in-Chief
JMIR Public Health and Surveillance

Dear Editor-in-Chief:

My colleagues and I would like to re-submit our revised manuscript entitled, "Multi-view child motor development dataset for AI-driven assessment of child development," for reconsideration as an original article in the GigaScience. This is the revised version of the previous manuscript (GIGA-D-22-00210).

First, we would like to thank the reviewers of the GigaScience for taking the time and effort to review our manuscript. The reviewers' comments were constructive and raised important and valuable points that provided us with some new insights about the study and required more clarification in the revised manuscript. Over the past few weeks, we have tried our best to improve our paper by attempting to fulfill all the reviewers' requirements. We have prepared detailed responses to the reviewers' comments. The modifications in the revised manuscript and supplemental material according to the reviewers' comments have been highlighted in blue characters. Additionally, we have thoroughly checked the grammatical errors in our manuscript again.

All authors have critically read and agreed to the re-submission of this manuscript. The paper is not under consideration elsewhere, and none of the paper's content has been published previously. In addition, no author of this manuscript has any relationship with the industry associated with the content of the manuscript.
Thank you in advance for the review.
Best regards,

Yu Rang Park, PhD
Associate Professor
Department of Biomedical Systems Informatics, Yonsei University College of Medicine, Yonsei University College of Medicine, 50-1 Yonsei-ro, Seodaemungu, Seoul, South Korea
Phone: +82-10-5240-3434
E-mail: yurangpark@yuhs.ac

Reviewer #1:

Concerns:
The authors claim that this is the first dataset of this kind to evaluate cognitive and motor functionality using AI.The paper is weak in its related works.
There are multiple papers that propose various tasks to assess embodied cognition in children using machine and deep learning.

Eg.

1. An automated assessment system for embodied cognition in children: from motion data to executive functioning
2. A multi-modal system to assess cognition in children from their physical movements
-focused to assess cognition with one physical movement
3. Motor assessment using the NIH Toolbox
4. Assessment of motor functioning in the preschool period
5. Detecting Children's Fine Motor Skill Development using Machine Learning
-focused on children's fine motor to implement classifiers (Fine model)
6. Deep learning assessment of child gross-motor

Specifically, papers 1,2,5,6 uses deep learning to evaluate motor functions in children.
Response: Thank you for informing us about the existing assessment tools that were lacking in our preliminary survey. We wanted to emphasize that our study is the first study to provide public access to the dataset. None of the previous studies mentioned in your comment have released their dataset. However, since motor skill evaluation using AI is valuable, the mentioned studies associated with the evaluation of motor functions were compared with our study in the Background section as follows.

"Concerning the use of artificial intelligence, various studies have evaluated children's motor functions—evaluation of cognition with physical movements[15,16], detection of machine learning-based fine motor skills[17], and evaluation of deep learning-based children's gross motor skills[18]—but they were all AI-based, model-oriented studies. Contrastingly, this study focused on presenting a dataset of children's gross motor skills for each age group."

Data collection:
The dataset consists of kids with healthy and specific conditions.Was there any correlation done between the kids with conditions and their performance to evaluate theeffectiveness of the task?
Response: Our dataset only included motor functioning in healthy children. Therefore, we did not need to perform correlations. However, it is meaningful to include children with specific conditions. We will consider it in our future work. We have added the following description in the Conclusion section.

"Additionally, the dataset will be extended to include children with and without developmental disabilities. It can be utilized to develop early diagnostic prediction models using AI techniques such as machine learning."

Question related to training.
How were the data split for the training and validation? Were they split based on the participants or based on samples?In computer vision, splitting the data based on samples might cause overfitting.
This is because,if a same kid is present in both training and validation set, its considered to memorize.

Response: Thank you for pointing out the unclear explanation. We split the data in 8(train):1(valid):1(test) ratio for the training and validation based on participants considering the overfitting problem as you have mentioned. We also obtained the loss curves after training, which showed that there was no overfitting problem. We have added loss graphs to Figure S1 of the supplement to show that there were no overfitting problems. Additionally, we have included a detailed description of the data split in Section 2.6 Evaluation for action recognition of the Method as follows.

"The dataset was split into three subsets based on the participants while considering the overfitting problem (see Supplement Figure S1): training (80%), validation (10%), and testing (10%). In age group A, the training, validation, and testing sets included 4,368 samples of 104 participants, 579 samples of 14 participants, and 593 samples of 13 participants, respectively. In age group B, the training, validation, and testing sets included 2,685 videos of 80 participants, 309 samples of 11 participants, and 360 samples of 8 participants, respectively. In age group C, the training, validation, and testing sets included 5049 samples of 125 participants, 687 samples of 17 participants, and 597 samples of 14 participants, respectively."

Training for 50 epochs seems too less for a video action recognition model. Were there any pre-training done?
Response: Thank you for your valuable question about our experiment setting. Although it was not mentioned in the manuscript, we trained for 100 epochs before training for 50 epochs because we found that our model had sufficiently converged before that point. Therefore, we did not pre-train the models. We have added the training graph with 100 epochs, including train accuracy and train loss to Figure S2 of the supplement for a better explanation of the epoch setting. We have described it in Section 2.6 Evaluation for action recognition of the Method as follows.

"Initially, we trained for 100 epochs to optimize the number of epochs for training. Since the models converged before 50 epochs, we trained for 50 epochs in the entire experiment (see Supplement Figure S2)."

So the Action recognition model can predict what action is being performed or even the score for the action?
Response: Thank you for seeking clarification on the action recognition model. The action recognition model can only predict actions being performed but not the score of the actions. Since the dataset disclosed by us includes scores (it is labeled), it can be used to develop a model for predicting scores in future work. We have added the following explanation to the Conclusion section.

"Since the dataset provided in this study includes scores, it can be used to develop a model for predicting scores. Furthermore, it can be utilized as the basis for developing screening tools for children's quantitative motor development levels (body maturity)."

How long was each recording? was a single recording split into multiple actions for the training and validation purpose?More details regarding the Model used, is required.
Response: Thank you for pointing out the insufficient description. The mean video length of age group A was 136 frames, the mean length of age group B was 167 frames, and the mean video length of age group C was 87 frames. We also obtained histograms of video lengths; video lengths were normally under 300 frames. Therefore, we set the maximum length of input data by 300 frames because the GCN-based action models that we used only accept inputs of the same length as RNNs. We padded it by zero if the sample length was shorter than 300 frames and sliced it to 300 frames if the sample length was longer than 300 frames. We did not split a single recording into multiple actions because each of our recordings contained only one trial for one kind of action. For the readers of this paper, we have added video length histograms to the supplement. Additionally, we have added the explanation of this process to Section 2.6 Evaluation for action recognition of the Method as follows.

"The mean length of data in age groups A, B, and C were 136, 167, and 87 frames, respectively. Videos were normally shorter than 300 frames based on the review of the video length histogram (see Supplement Figure S3). Therefore, the maximum length of input was set as 300 frames because the GCN-based action recognition models only accept inputs of the same length as RNNs. It was padded by zero if the sample length was shorter than 300 frames and sliced to 300 frames if the sample length was longer than 300 frames."

As this is a classification problem, a confusion matrix is required to get complete insight on the model performance.
Response: Thank you for letting us know that our results were insufficient. We have added the confusion matrices of three view models to Figure S4 of the supplement to provide a complete insight into the model performance, as per your valuable comment. The confusion matrix showed that models trained with three-view data were better than the models trained with front-view data only. We have added the explanation of confusion matrices to Section 3.2 Action recognition of the Result section as follows.

"Additionally, the confusion matrices of three-view models and single-view models were obtained (see Supplement Figure S4). The confusion matrices show that the diagonal of the three-view model's matrix

had higher values than the front-view model’s matrix. In other words, three-view models showed better performance than single-view models.”

Reviewer #2:

The study constructed a new dataset based on a skeleton of recordings of K-DST behaviors in children and validated the dataset using artificial intelligence model. In general, I think this work presents good research and has strong practical value. But this paper still has number of limitations. I suggest the authors make the following revisions.

Abstract
-p.2, Results: Why are the test accuracy results (…90%, 87.67%, and 95.45%...) inconsistent with the Section 3.2 Action recognition (Table 5)?
Response: Thank you for pointing our mistake. We have made corrections for consistent results in the Abstract.

Methods
-p.4: The acronym of "IRB" should be preceded by the full name.
Response: Thank you. We have added the full name of IRB as follows.

“This study was approved by the Institutional Review Board of Severance Hospital, Yonsei University College of Medicine, and the requirement for informed consent was waived (Institutional Review Board [IRB] number: 4-2021-0845).”

-p.5, section 2.2 Type of behavior, the second paragraph: What is the basis or principle for selecting the representative motor development behaviors for each age group? Please explain this.

Response: Thank you for your valuable comment.
As described in the methodology section of this paper, four core tasks were selected for each age group from a total of 48 GMS tasks provided by the K-DST. These core tasks were chosen by three pediatricians and 15 child behavior development experts based on the following three specific criteria. First, developmental milestones of each age group were considered in the selection of motor developmental behaviors, with the typical age at which these milestones are achieved identified from a 2010 study [24]. Second, physical and cognitive abilities were considered when selecting the core behaviors. Simple tasks, such as standing on one foot, were chosen for younger age groups with limited coordination abilities, whereas older age groups were given tasks that focused on coordination, such as stopping a rolling ball with one foot. Finally, actions that could measure various gross muscle functions were selected for each age group, including actions that involved the movement of the entire body, upper body, or lower body. Overall, the selection of representative motor development behaviors for each age group in this study was based on a combination of developmental milestones, physical and cognitive abilities, and actions that could measure various gross motor development functions.
For the readers of this paper, an explanation of this process has been added to Section 2.2 Type of behavior of the Method as follows.

“The principal criteria for selecting core tasks were: 1) developmental milestones, 2) physical and cognitive abilities, and 3) behaviors that measure various motor skills of each age group. First, developmental milestones were identified based on a 2010 pediatric review study [24]. Second, age-appropriate physical and cognitive abilities were considered. Simple tasks were selected for younger children with limited coordination, while coordination-based tasks were adopted for older children. Third, various gross motor functions were evaluated by examining the total muscle function through various movements involving the whole body, upper body, or lower body.”

-p.6, the title of Table 2: "…for four age groups…" should be "…for three age groups…".

Response: Thank you for pointing our mistake. We have revised it as per your correction.

-p.6, section 2.3 Experimental setup and data acquisition, the first paragraph: Why is the distance parameter different in different age groups (Figure 1B)? Is there any standard for setting this parameter?
Response: Thank you for pointing out the insufficient explanation. The distance from the camera for each age group was defined based on the child with the maximum height in the age group to measure the behavior of all children. We have added the following description in Section 2.3 Experimental setup and data acquisition of the Method section.

“To measure the behavior of all children, the distance from the camera for each age group was defined differently based on the child with the maximum height in each age group.”

-p.6, section 2.4 Annotation of behavior, the first paragraph: What is the role and content of the second stage review?
Response: Thank you for pointing out the insufficient explanation. We asked the same questions at both stages, but the pediatrician played a more confirmatory role. We also wanted to consider all perspectives of child development experts and pediatricians and increase the accuracy of evaluations with double reviews. We have added the following description in Section 2.4 Annotation of behavior of the Method section.

“The evaluation was conducted in two stages for three reasons. First, the opinions of pediatricians and child development experts were considered. Second, the assessments were double-checked to increase their accuracy. Third, the pediatricians’ role in the final stage was more confirmatory.”

Format
-Table 1-5: These tables can be presented in the format of "three-line table" because it is simple in form and easy to read.
-The format of the table should be consistent, such as the alignment, bold font (The header of Table 5).
-The text of this paper should be aligned at both ends.
Response: Thank you for detailed comments. We have made revisions based on your suggestions.

Reviewer #3: This paper presents a dataset of infants and children's activities used as criteria for assessing childhood development based on the Korean Development Screening Test (K-DST). The dataset comprises 399 infants and children. The age range is from 20 months to 71 months. The participants were grouped into three age groups: 20-35, 36-53, and 54-71 (younger, middle-aged, and older). The authors developed four criteria for assessing the motor skills of each group using the K-DST, following consultation with experts in the field.For data collection, three cameras were used to record the behavior of the participants 3-5 times. The footage was then annotated by experts to evaluate the behavior. After preprocessing the data, the dataset was analyzed using the deep learning model MS-G3D and the GCN-based action recognition model. The overall results demonstrated fairly good performance. This paper claims that the dataset is a valuable resource for creating AI algorithms
that assess children's behavior and track their development.

This is an interesting paper on the collection and analysis of a dataset focused on motor assessment of young children using camera-recorded gross motor activities. It is motivated by the need for easy, unbiased diagnostics that can be used to assess if children are suffering from motor delay, and by lack of activity data for young children, as current activity recognition models mostly focus on adults. In addition to the dataset, the paper includes an analysis of using pose estimation from the video to categorize children into year-and-a-half age groups.Overall, I like how this paper is written. It is concise, mostly clear, and covers a lot of the key points. I do have a few suggestions, which are described below.

In the first paragraph, there is mention that the most common clinical symptom of developmental disability is delayed acquisition of developmental technology. I think this sentence could be improved in a few ways:

- First, the phrasing is a little confusing to me because it sounds like the issue is a lack of access to

devices. I would use the term "developmental milestones" as stated earlier in the paper.
Response: Thank you for your insightful comment. We have revised it in the Background section as per your suggestion.

“Since a common clinical symptom of developmental milestones …”

- Second, I don't see strong citation support for this being the most common clinical symptom; the current citation is more an analysis of the demographics and specific factors that were observed in children who later received ASD diagnoses. I would agree that missed milestones is certainly the most prominent symptom since many parents pay attention to these, and the assessments are largely based on tasks associated with specific milestones. That said, there are other symptoms which could signal disability or delay, so I'd suggest including a reference that it is most common or adjusting the phrasing to be "a very common clinical symptom" or similar. It is a small change but could make the language more approachable to both experts and laypersons.
Response: We agree that there have been deficiencies as we have only emphasized ASD diagnoses. Based on your suggestion, we have revised the sentence.

- Third, on the point of citations, I think you could frame it as the need for catching developmental issues early because of (a) the association between motor development and later achievements like literacy and (b) the negative impact of delays on ability to start school and corresponding self-confidence issues. Below are a few citations you might want to consider to support these points; there are a number of relevant research studies which could further support the motivation if desired.

Duncan, Greg J., Chantelle J. Dowsett, Amy Claessens, Katherine Magnuson, Aletha C. Huston, Pamela Klebanov, Linda S. Pagani et al. "School readiness and later achievement." Developmental psychology 43, no. 6 (2007): 1428.

Burkam, David T., Laura LoGerfo, Doug Ready, and Valerie E. Lee. "The differential effects of repeating kindergarten." Journal of Education for Students Placed at Risk 12, no. 2 (2007): 103-136.

Broughman, Stephen P., and Mary R. Rollefson. "Children who enter kindergarten late or repeat kindergarten: Their characteristics and later school performance." Education Statistics Quarterly 2, no. 3 (2000): 21.

Suggate, Sebastian, Eva Pufke, and HeidrunStoeger. "Do fine motor skills contribute to early reading development?." Journal of Research in Reading 41, no. 1 (2018): 1-19.

Grissmer, David, Kevin J. Grimm, Sophie M. Aiyer, William M. Murrah, and Joel S. Steele. "Fine motor skills and early comprehension of the world: two new school readiness indicators." Developmental psychology 46, no. 5 (2010): 1008.

Response: Thank you for providing references to help us modify the need for early detection of developmental difficulties in a more understandable way. We have added the following sentences in the Introduction based on your recommended references.

“Additionally, early detection of developmental problems is crucial because delays can negatively affect a child’s readiness to start school. Furthermore, it can cause issues with self-confidence because it is associated with the child’s later achievements, such as literacy [3-5].”

About those last two: it's worth noting that they highlight the value of fine motor skill assessments in particular versus gross motor skill ones. While fine motor assessment is important, and I'd hope to see more on that in the future, I think there is definitely room to argue for the value of any motor assessments.I'm sure there is research support for that, which would be good to consider since this paper predominantly focuses on gross motor assessment.Along those same lines, it would be worth adding a small discussion about gross vs. fine motor skills. This wouldn't have to be extensive, but it could be part

of the motivation for why the gross motor portion of the KDST is used as the baseline as opposed to fine motor tasks. I'm not very familiar with the KDST, so perhaps it doesn't have as much of a fine motor section. I mention it because some of the research talks about fine motor being more critical for certain tasks, but as I said above, I do believe the gross motor assessment to be just as important with the right motivation!
Response: Thank you for highlighting the significance of assessing fine motor skills. Although we have concentrated on gross motor skills based on the result of the previous study that gross motor skills have more accuracy than fine motor in the K-DST, the importance of considering fine motor will be helpful in our future work. We have added the following in the Conclusion section.

"This study emphasized gross motor skills based on a previous study [7] that found the gross motor to have more accuracy than the fine motor in the K-DST for children's motor skill evaluation. However, other previous studies [3, 27] have shown that fine motor skills are also valuable in evaluating children's motor skills. In our future work, we will compare fine and gross motor skill evaluations to enhance the accuracy of child development evaluation."

Given the locality of the study, it's reasonable to use the KDST. If you wish, you could mention that other general assessments exist, like the Ages and Sages Questionnaire or the Bailey Mental Development Index. I don't feel it's necessary, but even a small mention that there are such assessments before introducing the KDST would be fine.
Response: Thank you for informing us about the existing assessment tools that were insufficient in our preliminary survey. We have added the following sentences in the Background section.

"Among several general development assessment tools for children such as such as the Ages and Stages Questionnaire [8,9], Bayley Mental Development Index [10], Bayley Scales of Infant Development, Wechsler Preschool and Primary Scale of Intelligence, and Peabody Developmental Motor Scales [11], the K-DST was selected because it can be assessed without money and has age-specific behaviors to assess motor development. In addition, recent K-DST-based research has demonstrated through national cohorts that the K-DST is a robust assessment of child development [7]."

How were the age groups selected for the dataset? That is, what was the process behind making Group A cover ages 20 to 35 months, Group B 36 to 53 months, and so on? I like these groupings and believe they align fairly well with key developmental stages, but I'm curious about the origin of the selection: something from the KDST, attempt to make the groups similarly sized, or something else. I often see these types of datasets use year-level scales, but I like the choice you made here.
Response: Precisely, our research team was concerned about recruiting an adequate number of children to evaluate gross motor skills through the AI model if the age group was too narrow. To address this concern, the K-DST target age of 4 to 71 months was divided into four age groups (4 to 19 months, 10 to 35 months, 36 to 53 months, and 54 to 71 months). However, due to the COVID-19 pandemic in Korea, fewer children were recruited than expected, especially in the youngest age group (4 to 19 months), with only 27 participants. Consequently, behavioral evaluation by AI models was difficult, and concerns about privacy violations arose if a group with too few participants was disclosed. Therefore, the age group of 4 to 19 months was excluded from the study to ensure that the size of each group was comparable and to avoid potential privacy concerns.

Likewise, the scoring mechanism of 0, 1, and 2: is this based on the standard scoring approach the experts would use? This 3-point scale seems reasonable, but it would be worth including the motivation for the selection versus something like a binary or 5-point scale.
Response: Thank you for your valuable comment. In this study, we presented a 3-point scale for gross motor scoring that modified the scoring criteria of the K-DST. The original K-DST evaluation scale is a 4-point scale. According to this scale, 0, 1, 2, and 3 points indicate not able to do at all, not able to do it, able to do it, and can do it well, respectively. Considering the original K-DST scoring system, there are almost no behaviors that received a score of 0 in our dataset. In order to match the similarity of data size between scores, we combined the original K-DST scores 0 and 1 and treated them as 0 points (bad), 2 as 1

(good), and 3 as 2 (perfect).
For the readers of this paper, an explanation of this process has been added to Section 2.4 Annotation of behavior of the Method as follows.

“This evaluation method utilized a 3-point scale, which is a modification of the 4-point scale used in the K-DST. The former regards 0 (not able to do at all) and 1 (not able to do it) in the 4-point scale as one score (0), 2 (able to do it) as 1, and 3 (can do it well) as 2.”

Table 5 uses Front, Left, and Right while the text uses front, x, and y. This could be remedied in the text by mentioning, even parenthetically, which is left and which is right after the angles of x and y are first mentioned.
Response: Thank you for correcting our mistake. We have revised it as “Front, Left, and Right” for consistency.

I'm not completely familiar with the method applied, so it would improve the readability to better explain how the combination of view angels worked. Based on the description, I'm imagining that one angle is fed frame-by-frame in into the machine learning system using pose-estimation coordinates. When combining views, are these concatenated so that all poses are sent at the same time, or are they interlaced in some manner?
Response: Thank you for your detailed question about our experiment. We did not consider how multi views were interlaced; however, in the results, the models trained with multi-view data showed better performance than models trained with only single-view data. It means that utilizing data from multi views had positive effects on the model training. However, it is essential to measure multi view’s actual effect, which we plan to measure in our future work. We have added the explanation of this future work to the Conclusion section as follows.

“Moreover, it was found that utilizing the multi-view data had positive effects on the model training. In our future work, we will measure the effect of multiple views by combining multiple data as an extended concept of multiple data utilization.”

Figure 2 caption mentions they are compiled into a list of joint coordinates, but it's not clear how this scale with multiple poses since the paper only discusses selecting a single pose from a frame.

Response: Thank you for pointing out the insufficient explanation. The list of coordinates mentioned in Figure 2 contains all the captured joint coordinates from only one view and not all the multi views. Initially, we had input the whole view at once and trained the models for each view separately as a feature extractor and combined the output features extracted from each model by a fully connected layer. However, the model did not converge and had overfitting problems. Therefore, we trained the models with data from each view independently. We have added the training graphs of the combination model in the Figure S5 of the supplement. For the readers of this paper, we have added a detailed explanation to Section 2.6 Evaluation for action recognition of the Method as follows.

“Therefore, there were 21 models: three age groups and seven view combination settings for each age group. Each model was trained with data including specific views depending on its view combination setting. Interconnections of multi views were not considered in the models. Models were trained with the data from each view independently.”

The results look good! I'd say the combination of all views worked best. I'm not sure if I'd say front individually is the most informative since it seems like the combination is what helps most. In any case, it's an impressive result, and even at the year-and-a-half level of granularity, it's a good showing of a possible motor skill diagnostic, mostly on gross motor tasks.

Given how much modern computer science paper bibliographies have expanded, I'm a little surprised to

see comparatively few citations. That said, I don't see an issue as long as it's enough to support everything stated or argued in the paper.

In conclusion, I really liked this paper! It's very direct and a pleasure to read. I do have a few suggestions above for ways make it more clear. I wouldn't say a lot needs to be changed since the paper is appropriately concise, just some clarifications and extra citations.

References

3. Grissmer, D., et al., Fine motor skills and early comprehension of the world: two new school readiness indicators. Dev Psychol, 2010. 46(5): p. 1008-17.
4. Kamphorst, E., et al., Emerging School Readiness Profiles: Motor Skills Matter for Cognitive- and Non-cognitive First Grade School Outcomes. Front Psychol, 2021. 12: p. 759480.
5. Duncan, G.J., et al., School readiness and later achievement. Dev Psychol, 2007. 43(6): p. 1428-1446.
7. Chung, H.J., et al., Development of the Korean Developmental Screening Test for Infants and Children (K-DST). Clin Exp Pediatr, 2020. 63(11): p. 438-446.
8. Romero Otalvaro, A.M., et al., ASQ-3: Validation of the Ages and Stages Questionnaire for the detection of neurodevelopmental disorders in Argentine children. Arch Argent Pediatr, 2018. 116(1): p. 7-13.
9. Veldman, S.L., et al., Prevalence and risk factors of gross motor delay in pre-schoolers. J Paediatr Child Health, 2020. 56(4): p. 571-576.
10. Bos, A.F., Bayley-II or Bayley-III: what do the scores tell us? Dev Med Child Neurol, 2013. 55(11): p. 978-9.
11. Zhang, Z., et al., Associations of sleep characteristics with cognitive and gross motor development in toddlers. Sleep Health, 2022. 8(4): p. 350-355.
15.Dillhoff, A., et al. An automated assessment system for embodied cognition in children: from motion data to executive functioning. in In Proceedings of the 6th international Workshop on Sensor-based Activity Recognition and Interaction. 2019.
16. Ramesh Babu, A., et al. A multi-modal system to assess cognition in children from their physical movements. in In Proceedings of the 2020 International Conference on Multimodal Interaction. 2020.
17. Polsley, S., et al., Detecting Children's Fine Motor Skill Development using Machine Learning. International Journal of Artificial Intelligence in Education, 2021(32): p. 991-1024.
18. Suzuki, S., Y. Amemiya, and M. Sato, Deep learning assessment of child gross-motor, in In 2020 13th International Conference on Human System Interaction (HSI). 2020, IEEE: Tokyo, Japan. p. 189-194.
24. Gerber, R.J., T. Wilks, and C. Erdie-Lalena, Developmental milestones: motor development. Pediatr Rev, 2010. 31(7): p. 267-76; quiz 277.
27. Suggate, S., E. Pufke, and H. Stoeger, Do fine motor skills contribute to early reading development? Journal of Research in Reading, 2018. 41(1): p. 1-19.

Close
