# Supplementary material for: Multiview child motor development dataset for AI-driven assessment of child development
Source: Gigascience. 2023 May 27;12:giad039. doi: 10.1093/gigascience/giad039 (PMC10220505; doi:10.1093/gigascience/giad039)
Supplement: giad039_Reviewer_1_Report_Original_Submission — Ashwin Ramesh Babu, Ph.D. -- 11/15/2022 Reviewed [file giad039_reviewer_1_report_original_submission.pdf]

**Reviewer Report**

**Title: Multi-view child motor development dataset for AI-driven assessment of child development**

**Version: Original Submission** **Date:** 11/15/2022

**Reviewer name: Ashwin Ramesh Babu, Ph.D.,**

**Reviewer Comments to Author:**

gigascienceThe paper proposes a dataset to assess motor function in children between the age of 21 to 70 monthsConcerns:The authors claim that this is the first dataset of this kind to evaluate cognitive and motor functionality using AI.The paper is weak in its related works.There are multiple papers that propose various tasks to assess embodied cognition in childrenusing machine and deep learning.Eg.1. An automated assessment system for embodied cognition in children: from motion data to executive functioning2. A multi-modal system to assess cognition in children from their physical movements3. Motor assessment using the NIH Toolbox4. Assessment of motor functioning in the preschool period5. Detecting Children's Fine Motor Skill Development using Machine Learning6. Deep learning assessment of child gross-motorSpecifically, papers 1,2,5,6 uses deep learning to evaluate motor functions in children.Data collection:The dataset consists of kids with healthy and specific conditions.were there any correlation done between the kids with conditions and their performance to evaluate theeffectiveness of the task?Question related to training.How were the data split for the training and validation? Were they split based on the participants or based on samplesIn computer vision, splitting the data based on samples might cause overfitting. This is because,if a same kid is present in both training and validation set, its considered to memorize.Training for 50 epochs seems too less for a video action recognition model. Were there any pre-training done?So the Action recognition model can predict what action is being performed or even the score for the action?How long was each recording? was a single recording split into multiple actions for the training and validation purpose?More details regarding the Model used, is required.As this is a classification problem, a confusion matrix is required to get complete insight on the model performance.

**Level of Interest**

Please indicate how interesting you found the manuscript: Choose an item.

**Quality of Written English**

Please indicate the quality of language in the manuscript: Choose an item.

**Declaration of Competing Interests**

Please complete a declaration of competing interests, considering the following questions:

- Have you in the past five years received reimbursements, fees, funding, or salary from an organisation that may in any way gain or lose financially from the publication of this manuscript, either now or in the future?
- Do you hold any stocks or shares in an organisation that may in any way gain or lose financially from the publication of this manuscript, either now or in the future?
- Do you hold or are you currently applying for any patents relating to the content of the manuscript?
- Have you received reimbursements, fees, funding, or salary from an organization that holds or has applied for patents relating to the content of the manuscript?
- Do you have any other financial competing interests?
- Do you have any non-financial competing interests in relation to this paper?

If you can answer no to all of the above, write 'I declare that I have no competing interests' below. If your reply is yes to any, please give details below.

I declare that I have no competing interests

I agree to the open peer review policy of the journal. I understand that my name will be included on my report to the authors and, if the manuscript is accepted for publication, my named report including any attachments I upload will be posted on the website along with the authors' responses. I agree for my report to be made available under an Open Access Creative Commons CC-BY license (http://creativecommons.org/licenses/by/4.0/). I understand that any comments which I do not wish to be included in my named report can be included as confidential comments to the editors, which will not be published.

Choose an item.

To further support our reviewers, we have joined with Publons, where you can gain additional credit to further highlight your hard work (see: https://publons.com/journal/530/gigascience). On publication of this paper, your review will be automatically added to Publons, you can then choose whether or not to claim your Publons credit. I understand this statement.

Yes Choose an item.
