# Supplementary material for: Multiview child motor development dataset for AI-driven assessment of child development
Source: Gigascience. 2023 May 27;12:giad039. doi: 10.1093/gigascience/giad039 (PMC10220505; doi:10.1093/gigascience/giad039)
Supplement: giad039_Reviewer_2_Report_Original_Submission — Lei Ma -- 1/16/2023 Reviewed [file giad039_reviewer_2_report_original_submission.pdf]

Reviewer Report

Title: Multi-view child motor development dataset for AI-driven assessment of child development

Version: Original Submission **Date:** 1/16/2023

Reviewer name: Lei Ma

Reviewer Comments to Author:

Dear authors,The study constructed a new dataset based on a skeleton of recordings of K-DST behaviors in children and validated the dataset using artificial intelligence model. In general, I think this work presents good research and has strong practical value. But this paper still has number of limitations. I suggest the authors make the following revisions.## Abstract-p.2, Results: Why are the test accuracy results (…90%, 87.67%, and 95.45%...) inconsistent with the Section 3.2 Action recognition (Table 5)?## Methods-p.4: The acronym of "IRB" should be preceded by the full name.-p.5, section 2.2 Type of behavior, the second paragraph: What is the basis or principle for selecting the representative motor development behaviors for each age group? Please explain this.-p.6, the title of Table 2: "…for four age groups…" should be "…for three age groups…".-p.6, section 2.3 Experimental setup and data acquisition, the first paragraph: Why is the distance parameter different in different age groups (Figure 1B)? Is there any standard for setting this parameter?-p.6, section 2.4 Annotation of behavior, the first paragraph: What is the role and content of the second stage review?## Format-Table 1-5: These tables can be presented in the format of "three-line table" because it is simple in form and easy to read.-The format of the table should be consistent, such as the alignment, bold font (The header of Table 5).-The text of this paper should be aligned at both ends.Best Regards

Level of Interest

Please indicate how interesting you found the manuscript: Choose an item.

Quality of Written English

Please indicate the quality of language in the manuscript: Choose an item.

Declaration of Competing Interests

Please complete a declaration of competing interests, considering the following questions:

- Have you in the past five years received reimbursements, fees, funding, or salary from an organisation that may in any way gain or lose financially from the publication of this manuscript, either now or in the future?
- Do you hold any stocks or shares in an organisation that may in any way gain or lose financially from the publication of this manuscript, either now or in the future?

- Do you hold or are you currently applying for any patents relating to the content of the manuscript?
- Have you received reimbursements, fees, funding, or salary from an organization that holds or has applied for patents relating to the content of the manuscript?
- Do you have any other financial competing interests?
- Do you have any non-financial competing interests in relation to this paper?

If you can answer no to all of the above, write 'I declare that I have no competing interests' below. If your reply is yes to any, please give details below.

I declare that I have no competing interests

I agree to the open peer review policy of the journal. I understand that my name will be included on my report to the authors and, if the manuscript is accepted for publication, my named report including any attachments I upload will be posted on the website along with the authors' responses. I agree for my report to be made available under an Open Access Creative Commons CC-BY license (http://creativecommons.org/licenses/by/4.0/). I understand that any comments which I do not wish to be included in my named report can be included as confidential comments to the editors, which will not be published.

Choose an item.

To further support our reviewers, we have joined with Publons, where you can gain additional credit to further highlight your hard work (see: https://publons.com/journal/530/gigascience). On publication of this paper, your review will be automatically added to Publons, you can then choose whether or not to claim your Publons credit. I understand this statement.

Yes Choose an item.
