# Supplementary material for: Multiview child motor development dataset for AI-driven assessment of child development
Source: Gigascience. 2023 May 27;12:giad039. doi: 10.1093/gigascience/giad039 (PMC10220505; doi:10.1093/gigascience/giad039)
Supplement: giad039_Reviewer_3_Report_Original_Submission — Tracy Anne Hammond -- 1/24/2023 Reviewed [file giad039_reviewer_3_report_original_submission.pdf]

Reviewer Report

Title: Multi-view child motor development dataset for AI-driven assessment of child development

Version: Original Submission **Date:** 1/24/2023

Reviewer name: Tracy Anne Hammond

Reviewer Comments to Author:

This paper presents a dataset of infants and children's activities used as criteria for assessing childhood development based on the Korean Development Screening Test (K-DST). The dataset comprises 399 infants and children. The age range is from 20 months to 71 months. The participants were grouped into three age groups: 20-35, 36-53, and 54-71 (younger, middle-aged, and older). The authors developed four criteria for assessing the motor skills of each group using the K-DST, following consultation with experts in the field.For data collection, three cameras were used to record the behavior of the participants 3-5 times. The footage was then annotated by experts to evaluate the behavior. After preprocessing the data, the dataset was analyzed using the deep learning model MS-G3D and the GCN-based action recognition model. The overall results demonstrated fairly good performance. This paper claims that the dataset is a valuable resource for creating AI algorithms that assess children's behavior and track their development.This is an interesting paper on the collection and analysis of a dataset focused on motor assessment of young children using camera-recorded gross motor activities. It is motivated by the need for easy, unbiased diagnostics that can be used to assess if children are suffering from motor delay, and by lack of activity data for young children, as current activity recognition models mostly focus on adults. In addition to the dataset, the paper includes an analysis of using pose estimation from the video to categorize children into year-and-a-half age groups.Overall, I like how this paper is written. It is concise, mostly clear, and covers a lot of the key points. I do have a few suggestions, which are described below.In the first paragraph, there is mention that the most common clinical symptom of developmental disability is delayed acquisition of developmental technology. I think this sentence could be improved in a few ways:- First, the phrasing is a little confusing to me because it sounds like the issue is a lack of access to devices. I would use the term "developmental milestones" as stated earlier in the paper.- Second, I don't see strong citation support for this being the most common clinical symptom; the current citation is more an analysis of the demographics and specific factors that were observed in children who later received ASD diagnoses. I would agree that missed milestones is certainly the most prominent symptom since many parents pay attention to these, and the assessments are largely based on tasks associated with specific milestones. That said, there are other symptoms which could signal disability or delay, so I'd suggest including a reference that it is most common or adjusting the phrasing to be "a very common clinical symptom" or similar. It is a small change but could make the language more approachable to both experts and laypersons.- Third, on the point of citations, I think you could frame it as the need for catching developmental issues early because of (a) the association between motor development and later achievements like literacy and (b) the negative impact of delays on ability to start school and corresponding self-confidence issues. Below are a few citations you might want to consider to support these points; there are a number of relevant research

studies which could further support the motivation if desired.Duncan, Greg J., Chantelle J. Dowsett, Amy Claessens, Katherine Magnuson, Aletha C. Huston, Pamela Klebanov, Linda S. Pagani et al. "School readiness and later achievement." Developmental psychology 43, no. 6 (2007): 1428.Burkam, David T., Laura LoGerfo, Doug Ready, and Valerie E. Lee. "The differential effects of repeating kindergarten." Journal of Education for Students Placed at Risk 12, no. 2 (2007): 103-136.Broughman, Stephen P., and Mary R. Rollefson. "Children who enter kindergarten late or repeat kindergarten: Their characteristics and later school performance." Education Statistics Quarterly 2, no. 3 (2000): 21.Suggate, Sebastian, Eva Pufke, and Heidrun Stoeger. "Do fine motor skills contribute to early reading development?." Journal of Research in Reading 41, no. 1 (2018): 1-19.Grissmer, David, Kevin J. Grimm, Sophie M. Aiyer, William M. Murrah, and Joel S. Steele. "Fine motor skills and early comprehension of the world: two new school readiness indicators." Developmental psychology 46, no. 5 (2010): 1008.About those last two: it's worth noting that they highlight the value of fine motor skill assessments in particular versus gross motor skill ones. While fine motor assessment is important, and I'd hope to see more on that in the future, I think there is definitely room to argue for the value of any motor assessments. I'm sure there is research support for that, which would be good to consider since this paper predominantly focuses on gross motor assessment.Along those same lines, it would be worth adding a small discussion about gross vs. fine motor skills. This wouldn't have to be extensive, but it could be part of the motivation for why the gross motor portion of the KDST is used as the baseline as opposed to fine motor tasks. I'm not very familiar with the KDST, so perhaps it doesn't have as much of a fine motor section. I mention it because some of the research talks about fine motor being more critical for certain tasks, but as I said above, I do believe the gross motor assessment to be just as important with the right motivation!Given the locality of the study, it's reasonable to use the KDST. If you wish, you could mention that other general assessments exist, like the Ages and Sages Questionnaire or the Bailey Mental Development Index. I don't feel it's necessary, but even a small mention that there are such assessments before introducing the KDST would be fine.How were the age groups selected for the dataset? That is, what was the process behind making Group A cover ages 20 to 35 months, Group B 36 to 53 months, and so on? I like these groupings and believe they align fairly well with key developmental stages, but I'm curious about the origin of the selection: something from the KDST, attempt to make the groups similarly sized, or something else. I often see these types of datasets use year-level scales, but I like the choice you made here.Likewise, the scoring mechanism of 0, 1, and 2: is this based on the standard scoring approach the experts would use? This 3-point scale seems reasonable, but it would be worth including the motivation for the selection versus something like a binary or 5-point scale.Table 5 uses Front, Left, and Right while the text uses front, x, and y. This could be remedied in the text by mentioning, even parenthetically, which is left and which is right after the angles of x and y are first mentioned.I'm not completely familiar with the method applied, so it would improve the readability to better explain how the combination of view angels worked. Based on the description, I'm imagining that one angle is fed frame-by-frame in into the machine learning system using pose-estimation coordinates. When combining views, are these concatenated so that all poses are sent at the same time, or are they interlaced in some manner? Figure 2 caption mentions they are compiled into a list of joint coordinates, but it's not clear how this scales with multiple poses since the paper only discusses selecting a single pose from a frame.The results look good! I'd say the combination of all views worked best. I'm not sure if I'd say front individually is the most informative since it seems like the combination is what helps most. In any case, it's an impressive

result, and even at the year-and-a-half level of granularity, it's a good showing of a possible motor skill diagnostic, mostly on gross motor tasks.Given how much modern computer science paper bibliographies have expanded, I'm a little surprised to see comparatively few citations. That said, I don't see an issue as long as it's enough to support everything stated or argued in the paper.In conclusion, I really liked this paper! It's very direct and a pleasure to read. I do have a few suggestions above for ways make it more clear. I wouldn't say a lot needs to be changed since the paper is appropriately concise, just some clarifications and extra citations.

Level of Interest

Please indicate how interesting you found the manuscript: Choose an item.

Quality of Written English

Please indicate the quality of language in the manuscript: Choose an item.

Declaration of Competing Interests

Please complete a declaration of competing interests, considering the following questions:

- Have you in the past five years received reimbursements, fees, funding, or salary from an organisation that may in any way gain or lose financially from the publication of this manuscript, either now or in the future?
- Do you hold any stocks or shares in an organisation that may in any way gain or lose financially from the publication of this manuscript, either now or in the future?
- Do you hold or are you currently applying for any patents relating to the content of the manuscript?
- Have you received reimbursements, fees, funding, or salary from an organization that holds or has applied for patents relating to the content of the manuscript?
- Do you have any other financial competing interests?
- Do you have any non-financial competing interests in relation to this paper?

If you can answer no to all of the above, write 'I declare that I have no competing interests' below. If your reply is yes to any, please give details below.

I declare that I have no competing interests

I agree to the open peer review policy of the journal. I understand that my name will be included on my report to the authors and, if the manuscript is accepted for publication, my named report including any attachments I upload will be posted on the website along with the authors' responses. I agree for my report to be made available under an Open Access Creative Commons CC-BY license (http://creativecommons.org/licenses/by/4.0/). I understand that any comments which I do not wish to

be included in my named report can be included as confidential comments to the editors, which will not be published.

Choose an item.

To further support our reviewers, we have joined with Publons, where you can gain additional credit to further highlight your hard work (see: https://publons.com/journal/530/gigascience). On publication of this paper, your review will be automatically added to Publons, you can then choose whether or not to claim your Publons credit. I understand this statement.

Yes Choose an item.
